# Measurement and genetic architecture of lifetime depression in the Netherlands as assessed by LIDAS (Lifetime Depression Assessment Self-report)

**DOI:** 10.1017/S0033291720000100

**Published:** 2021-06

**Authors:** Iryna O. Fedko, Jouke-Jan Hottenga, Quinta Helmer, Hamdi Mbarek, Floris Huider, Najaf Amin, Joline W. Beulens, Marijke A. Bremmer, Petra J. Elders, Tessel E. Galesloot, Lambertus A. Kiemeney, Hanna M. van Loo, H. Susan J. Picavet, Femke Rutters, Ashley van der Spek, Anne M. van de Wiel, Cornelia van Duijn, Eco J. C. de Geus, Edith J. M. Feskens, Catharina A. Hartman, Albertine J. Oldehinkel, Jan H. Smit, W. M. Monique Verschuren, Brenda W. J. H. Penninx, Dorret I. Boomsma, Mariska Bot

**Affiliations:** 1 Department of Biological Psychology, Vrije Universiteit Amsterdam, Amsterdam, The Netherlands; 2 Department of Epidemiology, Erasmus University Medical Center, Rotterdam, The Netherlands; 3 Department of Epidemiology and Biostatistics, Amsterdam University Medical Centres, location VUMC, Amsterdam, The Netherlands; 4 Amsterdam Public Health Research Institute, Amsterdam, The Netherlands; 5 Julius Center for Health Sciences and Primary Care, University Medical Center Utrecht, Utrecht, The Netherlands; 6 Department of Psychiatry, VU Medical Center, Amsterdam, The Netherlands; 7 Department of General Practice, Amsterdam University Medical Centres, Amsterdam, The Netherlands; 8 Radboud University Medical Center, Radboud Institute for Health Sciences, Nijmegen, The Netherlands; 9 Department of Psychiatry, Interdisciplinary Center Psychopathology and Emotion Regulation, University of Groningen, University Medical Center Groningen, Groningen, The Netherlands; 10 Centre for Nutrition, Prevention and Health Services, National Institute for Public Health and the Environment, Bilthoven, The Netherlands; 11 Division of Human Nutrition and Health, Wageningen University, Wageningen, The Netherlands; 12 Amsterdam Public Health and Amsterdam Neuroscience, Amsterdam, The Netherlands; 13 Department of Psychiatry, University of Groningen, University Medical Center Groningen, Groningen, The Netherlands; 14 Amsterdam UMC, Vrije Universiteit Amsterdam, Psychiatry, Amsterdam, The Netherlands

**Keywords:** LIDAS, Lifetime Depression Assessment Self-report, major depressive disorder, online assessment tool, prevalence

## Abstract

**Background:**

Major depressive disorder (MDD) is a common mood disorder, with a heritability of around 34%. Molecular genetic studies made significant progress and identified genetic markers associated with the risk of MDD; however, progress is slowed down by substantial heterogeneity as MDD is assessed differently across international cohorts. Here, we used a standardized online approach to measure MDD in multiple cohorts in the Netherlands and evaluated whether this approach can be used in epidemiological and genetic association studies of depression.

**Methods:**

Within the Biobank Netherlands Internet Collaboration (BIONIC) project, we collected MDD data in eight cohorts involving 31 936 participants, using the online Lifetime Depression Assessment Self-report (LIDAS), and estimated the prevalence of current and lifetime MDD in 22 623 unrelated individuals. In a large Netherlands Twin Register (NTR) twin-family dataset (*n* ≈ 18 000), we estimated the heritability of MDD, and the prediction of MDD in a subset (*n* = 4782) through Polygenic Risk Score (PRS).

**Results:**

Estimates of current and lifetime MDD prevalence were 6.7% and 18.1%, respectively, in line with population estimates based on validated psychiatric interviews. In the NTR heritability estimates were 0.34/0.30 (s.e. = 0.02/0.02) for current/lifetime MDD, respectively, showing that the LIDAS gives similar heritability rates for MDD as reported in the literature. The PRS predicted risk of MDD (OR 1.23, 95% CI 1.15–1.32, *R*^2^ = 1.47%).

**Conclusions:**

By assessing MDD status in the Netherlands using the LIDAS instrument, we were able to confirm previously reported MDD prevalence and heritability estimates, which suggests that this instrument can be used in epidemiological and genetic association studies of depression.

## Introduction

Major depressive disorder (MDD) is a common, complex mood disorder. Multiple factors of biological as well as environmental origin are affecting the risk to develop MDD (Otte et al., 2016). The 12-month and lifetime prevalence of MDD were estimated as 5.5% and 14.6% in high-income countries in the World Mental Health survey (Bromet et al., 2011). In line with global estimates, the 12-month and lifetime MDD prevalence were 5.2% and 18.7% in the Netherlands, respectively, based on Composite International Diagnostic Interview 3.0 (CIDI) (de Graaf, Ten Have, van Gool, & van Dorsselaer, 2012; Kessler & Üstün, 2004). The prevalence of MDD varies with socio-demographic factors (Kessler & Bromet, 2013). Females are more likely to develop MDD than males, the prevalence of both 12-month and lifetime MDD tends to change with age and individuals with a lower level of education are at higher risk of depression, as compared to those with a higher level of education (Bijl, Ravelli, & Van Zessen, 1998; Bromet et al., 2011; de Graaf et al., 2012).

Both genetic as well as environmental factors play a role in the liability to and the manifestation of MDD. Family studies find a significantly higher prevalence of MDD in biological relatives of MDD probands (Sullivan, Neale, & Kendler, 2000). Genetically informative studies have shown that this familial component is due to genetic factors rather than shared environmental risk. The meta-analytic heritability estimate of a depressive episode (according to ICF/ICD-10 classification) is 34% (Polderman et al., 2015). Heritability of lifetime MDD, estimated across four recall intervals in at least three interviews over a period of 9 years ranged between 34% and 41% with non-significant differences, indicating that the estimates do not depend on recall bias (Kendler & Aggen, 2001). Twin studies also looked into sex-specific heritability. Some studies reported no or little difference between sexes (Kendler & Prescott, 1999; Nivard et al., 2015), whereas some reported a higher heritability of lifetime MDD in women, than in men (Kendler, Gardner, Neale, & Prescott, 2001; Kendler, Gatz, Gardner, & Pedersen, 2006). However, there is no evidence for qualitative sex differences in the genetic architecture of depression with the empirical data indicating that the same genes are expressed in men and women (Eaves et al., 1997; Middeldorp, Wray, Andrews, Martin, & Boomsma, 2006; Vink et al., 2012).

International molecular genetic studies started to identify genetic variants associated with MDD risk after obtaining large sample sizes of hundreds of thousands individuals (Howard et al., 2018; Wray et al., 2018) with broad depression phenotype definitions. Single nucleotide polymorphisms (SNPs) summarized in Polygenic Risk Score (PRS) explain 1.9% of liability to MDD (Maier, Visscher, Robinson, & Wray, 2018; Wray et al., 2018). However, to obtain adequate power in these types of studies, large samples are needed. The power to detect associations could be reached with a smaller sample size when there is more homogeneity both in terms of population and definition of the depression phenotype (Cai et al., 2015; Mbarek et al., 2017). Such a large sample size and homogeneity are challenging to obtain, and usually Genome-Wide Association Studies (GWAS) meta-analyses of MDD show substantial heterogeneity in depression assessment between studies and the GWAS methods. One strategy that could yield homogeneous depression phenotypes while enabling large sample sizes is online phenotyping by a valid standardized instrument.

The Biobank Netherlands Internet Collaboration (BIONIC) project was started within the Biobanking and BioMolecular resources Research Infrastructure (BBMRI) in which the presence of MDD was established in large samples of individuals in a relatively homogeneous Dutch population with rich biomarker and ‘omics’ data. For this purpose, the Lifetime Depression Assessment Self-report (LIDAS) instrument was developed (see online Supplementary Material for the cohorts description and LIDAS questionnaire). LIDAS is an instrument for the assessment of MDD through self-report, which can be administered online or on paper. It follows the Diagnostic and Statistical Manual of Mental Disorders (DSM) criteria and is based on the CIDI short-form (Kessler, Andrews, Mroczek, Ustun, & Wittchen, 1998). The instrument showed moderate to good sensitivity (66–85%) and specificity (80–86%) in the validation study compared to depressed cases and controls as identified by the CIDI, and the lifetime MDD prevalence was estimated in a range from 19.6% to 20.8% depending on scoring algorithm, which is consistent with previous studies (Bot et al., 2017).

To evaluate whether MDD phenotype collected using a standardized online assessment tool (LIDAS) could be used in epidemiological and genetic association studies in this paper, we (1) characterize a large group of Dutch study participants taking part in the BIONIC project, in terms of MDD prevalence for a range of demographic characteristics, (2) report on the genetic architecture of MDD as assessed by LIDAS in a genetic and pedigree-based analysis of data from the Netherlands Twin Register (NTR) (Boomsma et al., 2018; Willemsen et al., 2013), (3) test to what extent PRS based on the recent Psychiatric Genomics Consortium (PGC) meta-analysis of genetic association studies of MDD predicts MDD risk in NTR (Wray et al., 2018). We compare the results to those obtained in large international studies to provide a ground for the future GWAS meta-analysis of LIDAS cohorts in the Netherlands.

## Methods

### MDD phenotype

Data were collected as part of a national collaboration to assess genetic variants of depression in Dutch cohorts. In 12 Dutch cohorts [the Netherlands Study of Anxiety and Depression (NESDA) (Penninx et al., 2008) pilot, NTR (Willemsen et al., 2013), Lifelines (Scholtens et al., 2015; Stolk et al., 2008), TRacking Adolescents' Individual Lives Survey (TRAILS) (Oldehinkel et al., 2015), TRAILS-CC (Clinical Cohort), Nijmegen Biomedical Study (NBS) (Galesloot et al., 2017), Nutrition Questionnaires plus (NQ plus)(Brouwer-Brolsma et al., 2018), Erasmus Rucphen Family (ERF) (Henneman et al., 2008), The Hoorn Diabetes Care System cohort (van der Heijden et al., 2017), The Hoorn Study (Rutters et al., 2017), The New Hoorn Study (Rutters et al., 2017), Doetinchem Study (Picavet, Blokstra, Spijkerman, & Verschuren, 2017; Verschuren, Blokstra, Picavet, & Smit, 2008)], the presence of lifetime and current (last year) depression was measured with the LIDAS. The LIDAS is a questionnaire, which includes the MDD screening questions according to DSM criteria. If a person did not respond positive to at least one of the two screening questions about the two core DSM MDD symptoms of depressed mood and loss of interest, then the section detailing other symptoms of MDD was not asked, in line with the DSM MDD criteria. This allows unaffected controls to fill out the questionnaire faster. The instrument further contained questions on diagnosis or treatment for depression or other mental disorders, sex, age, level of education, smoking, physical activity, weight, and height, to be completed by all participants. The instrument contains 41 questions in total (see online Supplementary Material).

Current (last year) and lifetime MDD phenotypes were scored according to DSM MDD criteria. MDD cases were defined as those fulfilling at least five of the nine DSM criteria, including one of the two core symptoms (depressed mood and/or loss of interest) and the rest out of seven symptoms (loss of energy, sleep problems, guilt/worthlessness, concentration problems/indecisiveness, psychomotor changes, weight or appetite change, suicidal ideation/thoughts of death), and replying positively to the question about serious interference with one's ability to do one's job, take care of household or family, or take care of oneself. Controls were defined as those who did not have any of the MDD core symptoms or less than five of the nine DSM MDD criteria or who answered negatively to the question about serious interference with one's ability to do one's job, take care of household or family, or take care of oneself. The instrument was validated against an interview (CIDI) in a subsample of individuals from NESDA (54% recruited via primary care) and NTR cohorts (Bot et al., 2017).

### Prevalence and demographic characteristics of participants

Eight population-based cohorts from the Netherlands were included in this study. Two cohorts (NESDA pilot and TRAILS-CC), in which the number of MDD cases was overrepresented due to study design, were excluded. The Hoorn Diabetes Care System cohort, The Hoorn Study, and The New Hoorn Study, in which persons with diabetes or pre-diabetes were oversampled, were combined into one Hoorn studies cohort. If information about family relatedness was available for cohorts (NTR), one person per family was randomly sampled.

We described the demographic characteristics such as sex, age, education, smoking status, physical activity, and body mass index (BMI) in each cohort and in the total sample. We categorized education into three levels (low, medium, and high) irrespective of whether diploma or certificate was received, with the exception of the high education group. If no diploma or certificate was received in that group, a participant would be included in the medium education group. For the Lifelines cohort, the question about education was slightly different and already assumed diploma or certificate being received and data were categorized taking it into account (online Supplementary Material). Age was classified into 18–39, 40–59, 60+ years. We inspected visually the distribution of age, height, weight, and BMI and excluded possible typos/outliers in age (120 years old, *N* = 1), height (<150 and >220 cm, *N* = 47), weight (<45 and >200 kg, *N* = 22), and BMI (<15 and >50, *N* = 25 after height and weight exclusion). Smoking status was represented by three groups, who answered to the question ‘Do you smoke?’ as (1) no, I never smoked; (2) I do not smoke at the moment, but I smoked in the past; and (3) yes. Physical activity was represented by three groups, namely by those who answered to the question ‘Do you at least once per week engage in physical activities in your leisure time that cause sweating?’ as (1) no; (2) 1x/2x per week; (3) 3x/4x per week or more. We categorized BMI (kg/m^2^) into four groups: underweight (<18.5); normal weight (18.5–24.9); overweight (25.0–29.9); obesity (⩾30.0). For some studies, the categorization resulted in small sample size per subgroup and such specific subgroups were excluded from analyses. For example, age group of 18–39 years was not represented in the Doetinchem and Hoorn Studies (*N* = 1) and groups of 40–59 and 60+ years were not represented in TRAILS (*N* = 0). The underweight subgroup was excluded from meta-analysis due to lack of observations in the majority of cohorts.

We first calculated the prevalence of MDD in each cohort. We assumed that true underlying effect (estimated proportion) is not the same across studies, because BIONIC cohorts differ between each other for a range of demographic characteristics. Therefore, we meta-analyzed results in a random-effects model in R package ‘meta’ using the inverse variance method (Schwarzer, 2007). Random-effects model means that estimated effect could differ due to unknown reasons (Serghiou & Goodman, 2019). We estimated the proportion of heterogeneity as quantified by *I*^2^ statistic, which ranges from 0% to 100% (Higgins & Thompson, 2002). We then performed the same analysis in subgroups according to the six demographic characteristics listed above (sex, age, education, smoking status, physical activity, and BMI). We tested whether differences between prevalence in each subgroup (e.g. low, medium, and high education) were statistically significant. We corrected for multiple testing using Bonferroni correction 0.05/12 = 4 × 10^−3^ taking into account two phenotypes (current and lifetime MDD) × 6 tests. We used meta-regression (Schwarzer, 2007) using subgroup strata (e.g. low, medium, and high education) as study-level covariates to explore the effect of the covariate on the amount of heterogeneity (*I^2^*) between cohorts. We tested the statistical significance of moderators using the omnibus test and QM test statistic as defined in R metafor package (Viechtbauer, 2010). Finally, we performed sensitivity analysis using a leave-one-out approach to identify influential cohorts.

To test whether BIONIC participants differ from the general population in terms of the prevalence of MDD and depression-related traits, we compared NTR participants that did participate in BIONIC and other NTR non-BIONIC participants in terms of depression-related traits such as (1) neuroticism, assessed by NEO Five-Factor Inventory (NEO-FFI) (Costa Jr & McCrae, 1989; McCrae & Costa Jr, 2007); (2) anxiety, assessed through the Anxious-Depressed scale of Adult Self Report (ASR) questionnaire (Achenbach & Rescorla, 2003); (3) depression, assessed through Hospital Anxiety and Depression Scale (HADS) (Zigmond & Snaith, 1983); and (4) answer to the question ‘Ever been in contact with counselling professionals for problems unrelated to physical health?’, which were available in NTR (online Supplementary Material).

### Heritability of LIDAS instrument in NTR

Within NTR, extended family data were collected allowing for genetically informed analysis to estimate narrow-sense heritability (*h*^2^, further in this paper, we will refer to it simply as heritability), that is the amount of phenotypic variation in case–control status that is accounted for by additive genetic factors (Boomsma et al., 2018; Willemsen et al., 2013). For these analyses, a strict definition of controls was applied by excluding individuals with self-reported diagnosis/treatment of any other psychiatric disorder.

We estimated heritability of current and lifetime MDD. To benchmark the analyses, we estimated the heritability of height and weight. Extended twin-family data were available for 18 838 individuals with age ⩾18 years. There were 13 868 (1390 cases and 12 478 controls) and 16 142 (3664 cases and 12 478 controls) individuals in whom the presence of current and lifetime MDD, respectively, could be ascertained. Data on self-reported height and weight were available for 18 113 and 18 102 individuals, respectively. Of these, individuals, who reported different sex (transgender, *N* = 4), whose sex or age was missing (*N* = 39), or who could not be linked to a pedigree because of ambiguous information on biological relatedness (*N* = 1) were excluded from all analyses.

For individuals with genotype data available, their ancestry was checked and non-Dutch participants were excluded (*N* = 351, see online Supplementary Material) (Abdellaoui et al., 2013). In total, 13 571 participants [1358 cases and 12 213 controls; 8399 pedigrees; 2345 monozygotic (MZ) and 2882 dizygotic (DZ) twin pairs] were included in the analysis of current MDD. Accordingly, 15 796 individuals (3583 cases and 12 213 controls; 9307 pedigrees; 2596 MZ and 3236 DZ twin pairs) were included in the estimation of lifetime MDD heritability. For height and weight, we additionally excluded possible typos/outliers outside of 5 standard deviations (s.d.) from the mean (*N* = 8 for height and *N* = 16 for weight). In total, 17 711 individuals (9954 pedigrees; 2805 MZ and 3518 DZ twin pairs) with data on height and 17 693 individuals (9941 pedigrees; 2803 MZ and 3514 DZ twin pairs) with data on weight ended up in these heritability analyses.

We estimated additive genetic (A), shared (C), and unique environmental (E) variance components in the Mendel software in a linear regression framework (Boomsma et al., 2018; Lange et al., 2013). Age and sex were added as fixed covariates. The additive genetic (A) variance relative to the total variance (the sum of A, C, and E) represents the heritability (*h*^2^). Note that in our data, C component was represented by environment shared by twins (both MZ and DZ) up till late adolescence (age 18) when they typically leave the parental household.

### MDD prediction based on PRS

With the increasing samples sizes in GWAS meta-analyses of MDD, more genetic makers, i.e. SNPs, have been identified as associated with MDD. When such marker effects are summarized in one score (Polygenic Risk Score, PRS profile) at an individual level, calculated as a sum of SNP risk alleles weighted by each risk allele effect identified in large MDD GWAS meta-analysis, it can be used to predict MDD in other datasets to assess its predictive power (Wray et al., 2014).

Within NTR, there were 4782 individuals (1078 cases and 3704 controls) with SNP and lifetime MDD data (see online Supplementary Material for the description of genotyping, imputation, and PRS calculation). For each individual, a PRS profile was computed based on summary statistics from the latest PGC GWAS meta-analysis of MDD in the individuals of European ancestry to date (Wray et al., 2018). The target dataset, the one on which the MDD phenotype will be predicted, was excluded from the discovery of GWAS meta-analysis, which was based on all other PGC cohorts. We computed PRS using LDpred software (Vilhjálmsson et al., 2015) assuming causal fraction of SNPs of 0.3 (see online Supplementary Material for details). To explore how well the PRS based on the broad definition of depression predicts LIDAS lifetime MDD, we (1) plotted the proportion of LIDAS MDD cases for each decile of PRS distribution; and (2) predicted LIDAS MDD case–control status of the participants in NTR from their corresponding PRS profiles. For the latter, we used generalized estimating equations with exchangeable correlation structure to account for relatedness within the dataset (Minică, Dolan, Kampert, Boomsma, & Vink, 2015). Age, sex, 10 principal components, and six genotyping chips were included as fixed covariates.

Finally, we estimated a genetic correlation between MDD as assessed by LIDAS and clinical MDD as assessed by CIDI, as estimated in the Mendel software package for pedigree analyses (Lange et al., 2013). Data on both these measurements were available in NTR and NESDA studies (see online Supplementary Material for details).

## Results

### Demographic and lifestyle characteristics

**Fig. 1.**
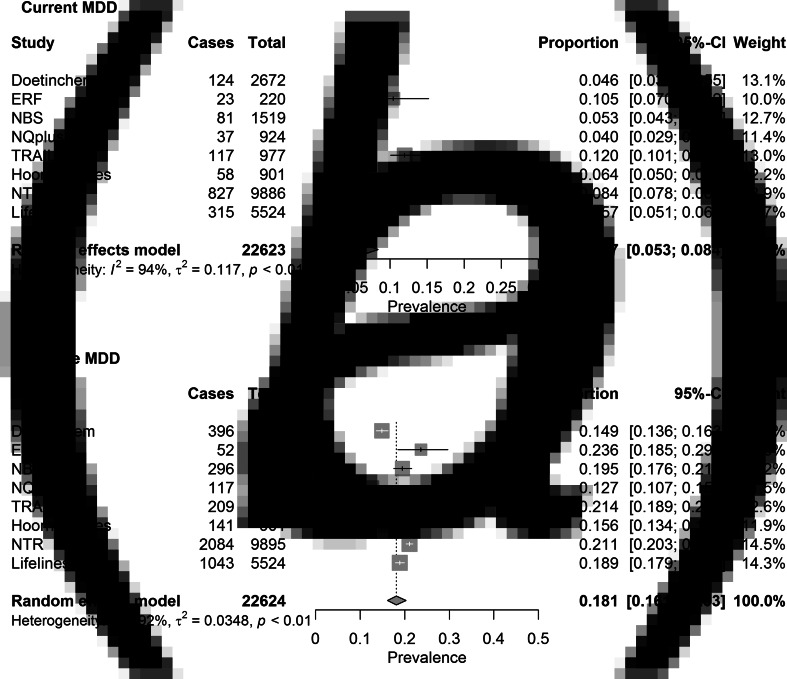
Pooled estimates of (*a*) current MDD and (*b*) lifetime MDD prevalence as measured with LIDAS.

The total number of participants reached 22 623 and 22 624 individuals for the current and lifetime MDD as defined by LIDAS. The total sample size per cohort slightly varies between current and lifetime MDD depending on whether last year episode item was filled in by a participant (Fig. 1). Cohort-specific number of participants and proportions of males/females, low/medium/high education, smoking status, physical activity, and mean (s.d.) of age and BMI are presented in Table 1. Proportion of males was smaller than the proportion of females in the majority of the cohorts and in the total sample, ranging from 33% in NTR to 49% in NBS, except for NQplus (56%) and The Hoorn Studies (60%). TRAILS was the youngest cohort with mean age 25.1 (s.d. = 0.6), whereas other cohorts mean age estimates ranged from 42.3 (s.d. = 16.3) in NTR to 68.1 (s.d. = 8.3) in The Hoorn Studies. Cohorts varied in the proportion of education level with the majority of participants falling into medium or high education groups. Most participants were either non-smoking or smoking in the past across cohorts, except in TRAILS, where the proportion of smokers was larger than that of non-smokers. Physical activity was distributed similarly across cohorts with most participants exercising 1–2 times per week (Table 1). The younger TRAILS cohort followed a different trend, where the proportion of those who exercise 3–4 times per week or more was similar to those who exercise 1–2 times per week (Table 1). BMI was similar across all cohorts (*M* total = 25.3, s.d. total = 1.0) with a somewhat increased mean estimate in The Hoorn Studies. Comparison of BIONIC respondents *v*. other participants in NTR showed that depression-related traits were similar in BIONIC NTR respondents and other NTR respondents (online Supplementary Material). Levels of anxiety, depression, neuroticism, and ‘contact with counselling professionals for problems unrelated to physical health’ were very similar across both groups (online Supplementary Material), indicating that participants in the BIONIC sample were not very different from other NTR participants, and thus may represent the general population.

**Table 1.** Demographic characteristics of BIONIC participants in whom lifetime MDD status could be determined^a^

	Doetinchem	ERF	NBS	NQplus	TRAILS	Hoorn Studies	NTR^b^	Lifelines^c^	Total
*N*	2663	220	1520	924	977	901	9895	5524	22 624
Sex, *N* (%)									
Males	1276 (48.2%)	106 (48.2%)	743 (49.0%)	515 (55.7%)	403 (41.2%)	538 (59.7%)	3307 (33.4%)	2275 (41.1%)	9163 (40.5%)
Females	1374 (51.8%)	114 (51.8%)	774 (51.0%)	409 (44.3%)	574 (58.8%)	363 (40.3%)	6588 (66.6%)	3258 (58.9%)	13 454 (59.5%)
Age, years									
Mean (s.d.)	65.6 (9.0)	55.6 (12.5)	63.2 (13.4)	58.7 (11.0)	25.1 (0.6)	68.1 (8.3)	42.3 (16.3)	55.4 (9.8)	50.7 (10.8)
*N*	2623	220	1517	924	977	899	9874	5533	22 567
Education, *N* (%)									
Low	600 (23.5%)	83 (38.1%)	198 (13.7%)	50 (5.5%)	13 (1.3%)	232 (27.5%)	588 (6.0%)	85 (1.6%)	1849 (8.4%)
Medium	1172 (45.8%)	88 (40.4%)	478 (33.1%)	316 (34.6%)	511 (52.3%)	399 (47.3%)	4817 (49.5%)	3133 (57.6%)	10 914 (49.3%)
High	785 (30.7%)	47 (21.6%)	768 (53.2%)	547 (59.9%)	453 (46.4%)	213 (25.2%)	4336 (44.5%)	2219 (40.8%)	9368 (42.3%)
Smoking, *N* (%)									
Never	1104 (41.7%)	91 (41.4%)	585 (38.5%)	458 (49.6%)	268 (27.7%)	292 (32.6%)	5602 (56.6%)	2501 (45.2%)	10 901 (48.2%)
Past smoker	1215 (45.9%)	105 (47.7%)	789 (51.9%)	418 (45.2%)	350 (36.2%)	530 (59.2%)	3162 (32.0%)	2296 (41.5%)	8865 (39.2%)
Current smoker	328 (12.4%)	24 (10.9%)	146 (9.6%)	48 (5.2%)	350 (36.2%)	74 (8.3%)	1127 (11.4%)	734 (13.3%)	2831 (12.5%)
Physical activity *N* (%)									
No	562 (21.4%)	67 (30.6%)	455 (30.0%)	162 (17.5%)	237 (24.8%)	325 (36.3%)	2150 (21.7%)	1155 (20.9%)	5113 (22.7%)
1/2 t.p.w.	1437 (54.7%)	114 (52.1%)	770 (50.7%)	528 (57.1%)	393 (41.2%)	397 (44.3%)	5254 (53.1%)	3001 (54.2%)	11 894 (52.7%)
3/4 or more t.p.w.	626 (23.8%)	38 (17.4%)	293 (19.3%)	234 (25.3%)	325 (34.0%)	174 (19.4%)	2488 (25.2%)	1377 (24.9%)	5555 (24.6%)
BMI									
Mean (s.d.)	26.1 (3.8)	26.7 (4.2)	25.3 (3.8)	25.0 (3.7)	24.0 (4.2)	28.6 (4.6)	24.6 (4.2)	26.0 (4.2)	25.3 (1.0)
*N*	2623	213	1488	909	954	884	9709	5516	22 296

t.p.w., times per week.

a For some individuals, sex, age, education, smoking, physical activity, or BMI were missing; however, they are still included in total prevalence calculation.

b Only unrelated individuals. The total sample size of NTR data with LIDAS assessment is 18 838.

c For Lifelines, demographic characteristics were calculated on the full data, including those for whom lifetime MDD status could not be determined.

### Prevalence

The pooled prevalence estimates of current and lifetime MDD in the total sample were 6.7% (95% CI 5.3–8.4%) and 18.1% (95% CI 16.1–20.3%), respectively (Fig. 1).

**Fig. 2.**
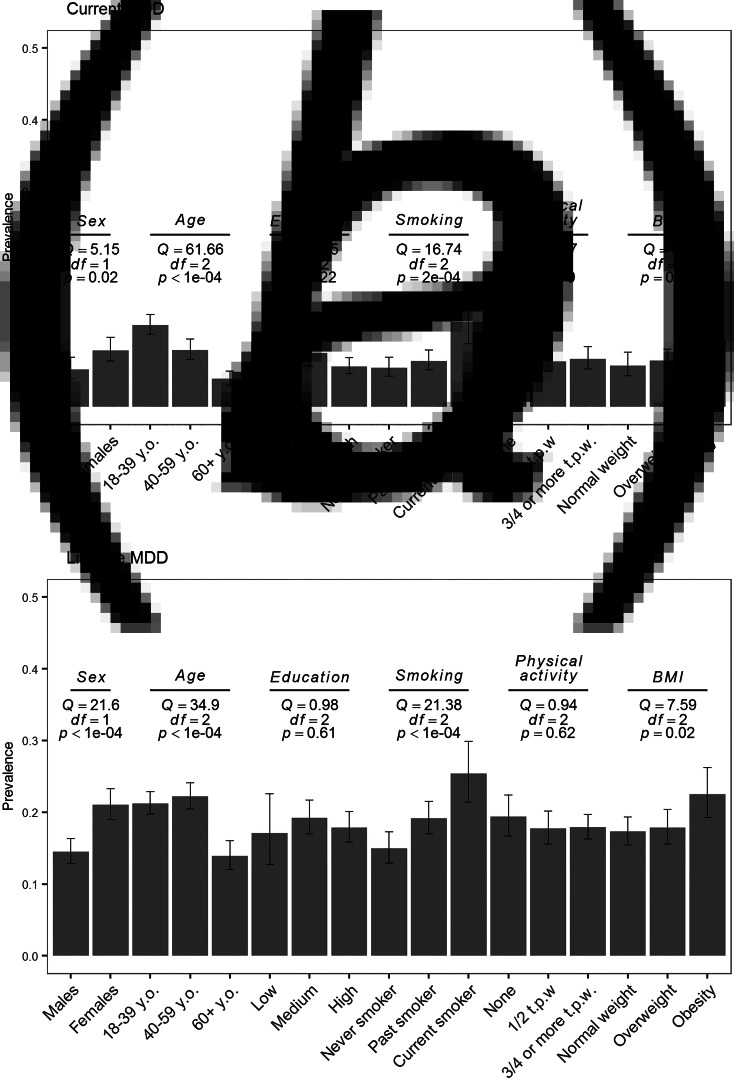
Pooled estimates of current (*a*) and (*b*) lifetime MDD prevalence as measured with LIDAS per subgroup (sex, age, education, smoking, physical activity, and obesity). *Q*, Cochran's *Q*; df, degrees of freedom; *p*, *p* value from the subgroup analysis of (*a*) current and (*b*) lifetime MDD; y.o., years old; t.p.w., times per week.

Prevalence estimates per strata are depicted in Fig. 2 and forest plots from the subgroup analyses are shown in online Supplementary Figs S2 and S3 for current and lifetime MDD, respectively. As expected, the prevalence of lifetime MDD was significantly lower in males (14.5%) than in females (21.0%). Prevalence of current MDD was following the same trend but this difference was not significant after correction for multiple testing (5.2% in males, 7.9% in females). Prevalence of both current and lifetime MDD was significantly lower in the age subgroup of 60 years and older (Fig. 2). We did not observe a significant difference in prevalence estimates in education level and physical activity strata for both current and lifetime MDD (Fig. 2). Estimates of prevalence were larger in smokers than in non-smokers with a significant difference between groups for both current and lifetime MDD. We observed a trend toward larger prevalence in the participants with obesity compared to participants with normal weight and overweight; however, it was not statistically significant for both current and lifetime MDD (Fig. 2).

The heterogeneity between studies was substantial for both current MDD [*I^2^* = 94% (95% CI 91–96%)] as well as for lifetime MDD [*I^2^* = 92% (95% CI 87–95%)]. We observed a reduced amount of heterogeneity between studies when data were stratified by sex and age groups (online Supplementary Figs S2 and S3), suggesting that part of the study heterogeneity for current and lifetime MDD depended on differences in samples in age and sex as suggested also by results of meta-regression.

Results of meta-regression showed that sex- and age-level covariates as defined by subgroups were significant contributors to heterogeneity between studies. Sex accounted for 24% [QM (df = 1) = 5.5, *p* = 0.02] and 72% [QM (df = 1) = 21.8, *p* < 0.0001] of heterogeneity for current and lifetime MDD. Age accounted for 80% [QM (df = 2) = 51.5, *p*⩽0.0001] and 75% [QM (df = 2) = 44.7, *p* < 0.0001] of variability between studies.

We performed a sensitivity analysis using leave-out one study at a time approach, which showed that no single study had a substantial influence on the meta-analysis results for current and lifetime MDD prevalence (online Supplementary Fig. S4).

### Heritability and PRS analyses

The estimated heritability of current and lifetime MDD were comparable and comprised 0.34 (s.e. = 0.02) and 0.30 (s.e. = 0.02), respectively. To benchmark the analysis, we report heritability estimates of height and weight, which were 0.81 (s.e. = 0.009) and 0.55 (s.e. = 0.01). Estimates of A, C (shared environment for twins), and E variance components with their standard errors (s.e.) are reported for each phenotype in online Supplementary Table S1. All variance components were significantly different from zero for all traits, except the C component for current and lifetime MDD, suggesting that twins shared environment does not contribute to the variation in MDD status. The direction of sex effect was negative for males (−0.05 for lifetime and −0.02 for current MDD) and positive for females (0.05 for lifetime and 0.02 for current MDD), indicating a higher MDD risk for females.

**Fig. 3.**
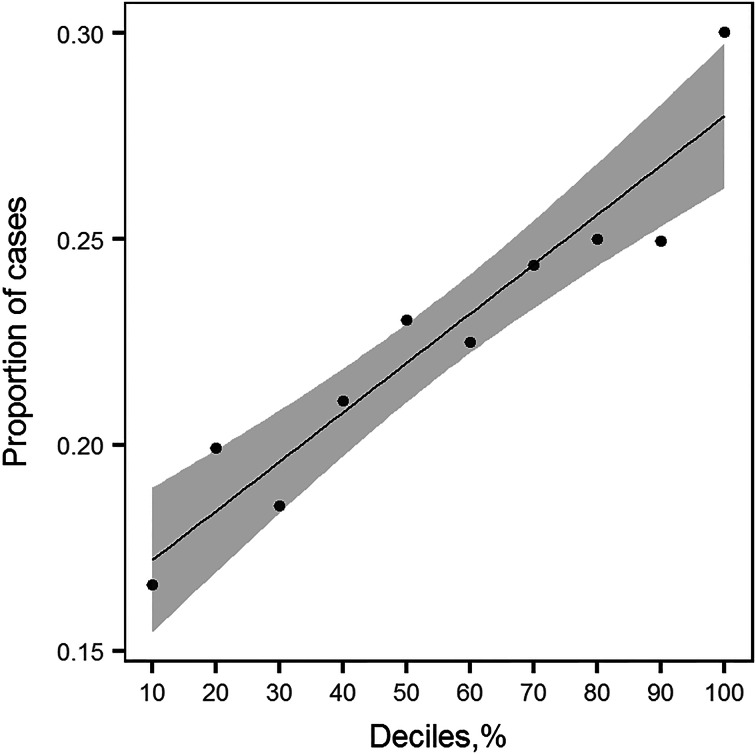
Proportion of lifetime MDD cases in NTR plotted against deciles of PRS profiles distribution. For each group of individuals falling into *i*^th^ decile of PRS distribution, the proportion of cases was calculated. This plot shows the relationship between the increasing value of PRS profile and the increased number of MDD cases. The line plotted through the points is a linear regression line with shaded area around it depicting the 95% confidence interval.

We observed a positive linear relationship between deciles based on the PRS distribution and the proportion of lifetime MDD cases (Fig. 3). The proportion of cases increases linearly with an increase of PRS, indicating that the group of people with higher PRS have a larger proportion of MDD cases as defined with the LIDAS (Fig. 3). The PRS significantly predicted lifetime MDD case/control status (OR 1.23, 95% CI 1.15–1.32). Pseudo-*R*^2^ indicating the proportion of variance explained in the phenotype by the PRS was 1.20%, and 1.47% on liability scale (Lee, Goddard, Wray, & Visscher, 2012).

The number of participants with both LIDAS and CIDI data was 1682 individuals (186 cases and 1496 controls for LIDAS and 68 cases and 1614 controls for CIDI MDD, see online Supplementary Table S2). The time interval between assessment by CIDI and by LIDAS was substantial. Cases in NTR and NESDA were assessed about 9 years before LIDAS (Middeldorp et al., 2006; Sullivan et al., 2009), thus recall bias may explain a change in status. Controls in NESDA were identified as scoring with no depression on all CIDI assessments, including the most recent one a few months before completing LIDAS. The genetic correlation between MDD assessed by LIDAS and by CIDI was 0.70.

## Discussion

In the current study, we addressed a question of whether MDD phenotype collected using a standardized online assessment tool (LIDAS) could be used in epidemiological and genetic association studies. The instrument was validated against a diagnostic interview in a previous study (Bot et al., 2017). Here we reported the prevalence of current and lifetime MDD in eight Dutch cohorts involving 22 623 participants, and explored the genetic architecture of MDD as established by the LIDAS. We investigated whether the prevalence of current and lifetime MDD differed according to sex, age, education, smoking status, physical activity, and BMI.

Prevalence of current MDD in BIONIC was estimated as 6.7% (95% CI 5.3–8.4%) for the total sample and as 5.2% (95% CI 3.9–6.9%) for males and 7.9% (95% CI 6.4–9.7%) for females. This is in line with previous studies conducted in the Netherlands, where 12-month MDD prevalence was 5.8% with estimates for males as 4.1% and for females as 7.5% (Bijl et al., 1998). A study published about 14 years later showed similar estimates for the total sample (5.2%), as well as for males (4.1%) and females (6.3%) (de Graaf et al., 2012). Lifetime MDD prevalence in our data was estimated as 18.1% (95% CI 16.1–20.3%) with significantly different estimates for males (14.5%, 95% CI 12.9–16.3%) and females (21.0%, 95% CI 19.0–23.3%). Lifetime MDD prevalence estimates were in line with the estimate found in the two previous studies in the Netherlands (overall prevalence 15.4/18.7%, prevalence in males 10.9/13.1%, and prevalence in females 20.1/24.4%) (Bijl et al., 1998; de Graaf et al., 2012). With our data, we largely confirmed previously reported socio-demographic and lifestyle correlates of MDD. Previous studies indicated that the current MDD prevalence decreases with age (Bijl et al., 1998; de Graaf et al., 2012; Ohayon, 2007), which we confirmed in our study. For lifetime MDD, we observed that the prevalence is similar in middle-aged and younger adults, but is much lower in the age subgroup of 60+. This has been reported before, but is counter intuitive as depressive episodes are likely to accumulate in life, and could indicate recall bias, selective survival, or a cohort effect (Andrade et al., 2000; Kessler et al., 2007, 2010). Significant difference in prevalence between males and females, different age groups, and effect of these variables on heterogeneity between studies warrants further sex- and age-specific research as different etiological factors could be involved in liability to MDD in these subgroups. We observed a trend of increased prevalence of both current and lifetime MDD in relation to smoking and higher BMI, which is in line with previous research (Fergusson, Goodwin, & Horwood, 2003; Glassman et al., 1990; Ohayon, 2007; Weinberger et al., 2017). Note that the association between increased MDD prevalence and higher BMI could be driven by The Hoorn Studies, which are represented by older participants and include type 2 diabetes patients. We found similar current and lifetime MDD prevalence in education and physical activity strata, although there is a trend to a lower MDD prevalence in higher education group, which has been shown in previous research (Andrade et al., 2000; Ohayon, 2007).

MDD is a common and multifactorial disorder with many genetic and environmental factors contributing to the risk. The amount of additive genetic variation that contributes to the variation in liability to MDD (heritability, *h*^2^) is an important population characteristic of the phenotype. Heritability of current and lifetime MDD in our data were 0.34 (s.e. = 0.02) and 0.30 (s.e. = 0.02), respectively. This is in line with the meta-analytic estimate based on previous studies of twin data (*h*^2^ = 34%) (Polderman et al., 2015). To benchmark the analysis, we estimated heritability for height and weight, which were highly consistent with previous research (Dubois et al., 2012; Elks et al., 2012; Silventoinen et al., 2003). PRS profiles of NTR individuals computed based on the results of an international GWAS meta-analysis using a rather broad definition of MDD were significantly associated with the risk of lifetime MDD as assessed by LIDAS, although the amount of variation explained by PRS profile on liability scale was somewhat lower in LIDAS (*R*^2^ = 1.47%) than in the GWAS meta-analysis (*R*^2^ = 1.9%) (Wray et al., 2018). In addition, the results of the genetic correlation analysis suggested that similar genetic factors are involved to a large extent (*r*_g_ = 0.70) between MDD assessed by LIDAS and CIDI. Note, however, that the sample size was not large (*N* total = 1682 persons) and that there was a 9-year interval between the two assessments.

In conclusion, the similarity of MDD prevalence and genetic architecture in BIONIC as compared to other Dutch general population studies of MDD suggests that the BIONIC data assessed with LIDAS are suitable to use in future epidemiological and genetic studies of MDD. Studying etiological factors of MDD is often difficult and costly. With the introduction of the LIDAS, more opportunities for the assessment of MDD in the context of other health-related or demographic studies become available and this project contributes to the opportunities to collect and need of increased sample size that is needed for a better understanding of the biological and genetic aspects of MDD.

## Supplementary material

For supplementary material accompanying this paper visit https://doi.org/10.1017/S0033291720000100. click here to view supplementary material
