# Supplementary material for: Measurement and genetic architecture of lifetime depression in the Netherlands as assessed by LIDAS (Lifetime Depression Assessment Self-report)
Source: Psychol Med. 2020 Feb 27;51(8):1345–54. doi: 10.1017/S0033291720000100 (PMC8223240; doi:10.1017/S0033291720000100)
Supplement: Supplementary file 1 [file S0033291720000100sup001.docx]

**Measurement and genetic architecture of lifetime depression in the Netherlands**

**as assessed by LIDAS (Lifetime Depression Assessment Self-report)**

Iryna O. Fedko^1^, Jouke-Jan Hottenga^1^, Quinta Helmer^1^, Hamdi Mbarek^1^, Floris Huider^1^, Najaf Amin^2^, Joline W. Beulens^3,4,5^, Marijke A. Bremmer^6^, Petra J. Elders^4,7^, Tessel E. Galesloot^8^, Lambertus A. Kiemeney^8^, Hanna M. van Loo^9^, H. Susan J. Picavet^10^, Femke Rutters^3,4^, Ashley van der Spek^2^, Anne M. van de Wiel^11^, Cornelia van Duijn^2^, Eco J.C. de Geus^1,12^, Edith J.M. Feskens^11^, Catharina A. Hartman^13^, Albertine J. Oldehinkel^13^, Jan H. Smit^14^, W. Monique M. Verschuren^5,10^ , Brenda W.J.H. Penninx^12,14^, Dorret I. Boomsma^1,12^, and Mariska Bot^12,14^

^1^ Department of Biological Psychology, Vrije Universiteit Amsterdam, The Netherlands

^2^ Department of Epidemiology, Erasmus University Medical Center, Rotterdam, The Netherlands

^3^ Department of Epidemiology and Biostatistics, Amsterdam University Medical Centres, location VUMC, The Netherlands

^4^ Amsterdam Public Health Research Institute, The Netherlands

^5^ Julius Center for Health Sciences and Primary Care, University Medical Center Utrecht, Utrecht, The Netherlands

^6^ Department of Psychiatry, VU Medical Center, Amsterdam, The Netherlands

^7^ Department of General Practice, Amsterdam University Medical Centres, The Netherlands

^8^ Radboud university medical center, Radboud Institute for Health Sciences, Nijmegen, The Netherlands

^9^ Department of Psychiatry, Interdisciplinary Center Psychopathology and Emotion Regulation, University of Groningen, University Medical Center Groningen, The Netherlands

^10^ Centre for Nutrition, Prevention and Health Services, National Institute for Public Health and the Environment, Bilthoven, The Netherlands

^11^ Division of Human Nutrition and Health, Wageningen University, Wageningen, The Netherlands

^12^ Amsterdam Public Health and Amsterdam Neuroscience, The Netherlands

^13^ Department of Psychiatry, University of Groningen, University Medical Center Groningen, Groningen, Netherlands

^14^ Amsterdam UMC, Vrije Universiteit Amsterdam, Psychiatry, Amsterdam, The Netherlands

Table of Contents

[Description of the Dutch cohorts participated in BIONIC project 3](#_Toc27046486)

[BIONIC cohort acknowledgements and financial support 6](#_Toc27046487)

[Genotyping, imputation and PCA in NTR 10](#_Toc27046488)

[Polygenic Risk Score (PRS) analysis in NTR 10](#_Toc27046489)

[Comparison of BIONIC respondents and other cohort participants for personality traits and/or other depression related phenotypes 12](#_Toc27046490)

[Supplementary Figure 1. 13](#_Toc27046491)

[Cut-off for low, medium and high education levels of BIONIC cohorts* 14](#_Toc27046492)

[Genetic correlation analyses 15](#_Toc27046493)

[LIDAS questionnaire 16](#_Toc27046494)

[Supplementary Figure 2. 20](#_Toc27046497)

[Supplementary Figure 3. 26](#_Toc27046498)

[Supplementary Figure 4. 32](#_Toc27046499)

[Supplementary Table 1. 33](#_Toc27046500)

[Supplementary Table 2. 33](#_Toc27046501)

[References: 34](#_Toc27046502)

# Description of the Dutch cohorts participated in BIONIC project

*Doetinchem Cohort Study*

In the Doetinchem Cohort Study (DCS), men and women selected randomly from the general population and aged 20-59 years at inclusion in 1987-1991, are assessed every five years with the 7th measurement round running between 2018 and 2022([Picavet, Blokstra, Spijkerman, & Verschuren, 2017](#_ENREF_17); [Verschuren, Blokstra, Picavet, & Smit, 2008](#_ENREF_25)). A broad range of lifestyle, biological, health (physical and psycho-social), wellbeing, disease and care factors are measured. The protocols for subsequent rounds were approved by the Medical Ethical Committee of TNO (rounds 2 and 3), respectively the Medical Ethical Committee of University Medical Center Utrecht (rounds 4-7). All participants gave written informed consent.

*Erasmus Rucphen Family (ERF)*

ERF is a family-based study that includes inhabitants of a genetically isolated community in the South-West of the Netherlands, ascertained as part of the Genetic Research in Isolated Population (GRIP) program. The ERF cohort includes approximately 3000 living descendants of 22 founder couples who had at least six children baptized in the community church. The population shows minimal immigration and high inbreeding. All participants provided informed consent. The Medical Ethical Committee of the Erasmus University Medical Centre approved the ERF study ([Pardo, MacKay, Oostra, van Duijn, & Aulchenko, 2005](#_ENREF_15)).

*The Hoorn Diabetes Care System cohort*

The Hoorn Diabetes Care System cohort (DCS) is a prospective cohort representing a comprehensive dataset on the natural course of T2D, with repeated yearly clinical measures and outcomes. The DCS consists of persons with T2D in primary care from the West-Friesland region of the Netherlands. Enrolment in the cohort started in 1998 and this prospective dynamic cohort currently holds 12 673 persons with T2D. A subgroup of 600 participants was approached for additional measurements in 2016, including completing the LIDAS questionnaire. All participants gave written informed consent ([van der Heijden et al., 2017](#_ENREF_24)).

*The Hoorn Study*

In the year 1989, the Hoorn Study was initiated to study the prevalence and risk factors of impaired glucose metabolism and type 2 diabetes in a Western European population. This initially cross-sectional study has been extended to a prospective cohort over the past decades, to study the complications associated with disturbances in glucose metabolism and diabetes care. At baseline this cohort consisted of 2484 participants, which were aged on average 61.7 years. A subgroup of 71 participants consisting of participants with normal glucose tolerance, prediabetes and T2D was approached for additional measurements in 2016, including completing the LIDAS questionnaire. All participants gave written informed consent ([Rutters et al., 2017](#_ENREF_21)).

*The New Hoorn Study*

In the year 2006, a second, similar cohort was initiated, called the New Hoorn Study. The design was based on the design of the earlier Hoorn Study. The primary objective now was to investigate whether the increasing rates of longevity, physical inactivity and obesity affect the prevalence and risk factors of disturbances in glucose metabolism. At baseline this cohort consisted of 2807 participants, which were aged on average 53.4 years. A subgroup of 235 participants consisting of participants with prediabetes was approached for additional measurements in 2016, including completing the LIDAS questionnaire. All participants gave written informed consent ([Rutters et al., 2017](#_ENREF_21)).

*Lifelines*

Lifelines is a multi-disciplinary prospective population-based cohort study examining in a unique three-generation design the health and health-related behaviours of 167,729 persons living in the North of The Netherlands. It employs a broad range of investigative procedures in assessing the biomedical, socio-demographic, behavioural, physical and psychological factors which contribute to the health and disease of the general population, with a special focus on multi-morbidity and complex genetics.

The Lifelines Biobank initiative has been made possible by subsidy from the Dutch Ministry of Health, Welfare and Sport, the Dutch Ministry of Economic Affairs, the University Medical Center Groningen (UMCG, the Netherlands), University Groningen and the Northern Provinces of the Netherlands.

*Netherlands Twin Register (NTR)*

The Netherlands Twin Register (NTR) is a population based study, which longitudinally collects data about health and behavior of twins and their families ([van Beijsterveldt et al., 2013](#_ENREF_23); [Willemsen et al., 2013](#_ENREF_27)). Informed consent was obtained from all participants. The study was approved by the Central Ethics Committee on Research Involving Human Subjects of the VU University Medical Centre, Amsterdam, an Institutional Review Board certified by the U.S. Office of Human Research Protections (IRB number IRB00002991 under Federal-wide Assurance- FWA00017598; IRB/institute codes, NTR 03-180).

*Nijmegen Biomedical Study (NBS)*

The Nijmegen Biomedical Study (NBS) is a population-based survey conducted by the Department for Health Evidence and the Department of Laboratory Medicine of the Radboud university medical center. Details of the study have been described before ([Galesloot et al., 2017](#_ENREF_9)). Briefly, 22,451 age and sex stratified randomly selected inhabitants of the municipality of Nijmegen received an invitation to fill out a postal questionnaire on, e.g., lifestyle and medical history, and to donate an 8.5 ml blood sample in a serum separator tube and a 10 ml EDTA blood sample. The response to the questionnaire was 42% (N=9350). 69% (N=6468) of the responders donated blood samples.

*Nutrition Questionnaires plus (NQplus)*

The NQplus study is a prospective cohort study among 2048 Dutch men (52%) and women (48%) aged 20-70 years ([Brouwer-Brolsma et al., 2018](#_ENREF_4)). At baseline we assessed habitual dietary intake, conducted physical examinations, collected blood and 24 hour urine and administered a variety of validated questionnaires. All measurements were repeated after 1 and 2 years of follow-up.

*TRacking Adolescents’ Individual Lives Survey (TRAILS)*

The TRacking Adolescents’ Individual Lives Survey (TRAILS) is a population-based study, in which 2230 initially 10-12-year-olds have been followed from 2001 onwards ([Oldehinkel et al., 2015](#_ENREF_14)). The LIDAS was administered when the participants were, on average, 25.7 (SD = 0.6) years old. The study was approved by Dutch Central Committee on Research Involving Human Subjects (CCMO), participants were treated in accordance with the Declaration of Helsinki, and written consent was acquired from all adolescents and their parents.

# BIONIC cohort acknowledgements and financial support

*Doetinchem Cohort Study*

The Doetinchem Cohort Study is supported by the Dutch Ministry of Health, Welfare and Sport and the National Institute for Public Health and the Environment. The team of the Doetinchem Cohort Study thanks the respondents, epidemiologists, and fieldworkers of the Municipal Health Service in Doetinchem for their contribution to the data collection for this study.

*Erasmus Rucphen Family (ERF)*

The ERF study as a part of EUROSPAN (European Special Populations Research Network) was supported by European Commission FP6 STRP grant number 018947 (LSHG-CT-2006-01947) and also received funding from the European Community's Seventh Framework Programme (FP7/2007-2013)/grant agreement HEALTH-F4-2007-201413 by the European Commission under the programme "Quality of Life and Management of the Living Resources" of 5th Framework Programme (no. QLG2-CT-2002-01254). We are grateful to all study participants and their relatives, general practitioners and neurologists for their contributions and to P. Veraart for her help in genealogy, J. Vergeer for the supervision of the laboratory work and P. Snijders for his help in data collection.

*The Hoorn Diabetes Care System cohort*

To perform additional research in (subsamples of) the DCS cohort, funding has been received from several instances including the VUMC, Dutch Federation of University Medical Centres, health insurers, Dutch Science Organisation NWO, Dutch Organization for Health Research and Development ZonMw, Dutch Diabetes Foundation, European Foundation for the Study of Diabetes, International Diabetes Federation, European Innovative Medicine Initiative and European Union.

*The Hoorn Study*

In past years, funding for the Hoorn Study was provided by several different sponsors at certain points in time, including: the VU University Medical Center of Amsterdam, the Netherlands Organization for Scientific Research (NWO), the Netherlands Organization for Health Research and Development (ZonMW), the Dutch Diabetes Research Foundation and the Netherlands Heart Foundation.

*The New Hoorn Study*

Funding for the New Hoorn Study was provided by: the VU University Medical Center of Amsterdam, Novartis Pharma B.V, the European Union and the Innovative Medicine Initiative.

*Lifelines*

The Lifelines Biobank initiative has been made possible by subsidy from the Dutch Ministry of Health, Welfare and Sport, the Dutch Ministry of Economic Affairs, the University Medical Center Groningen (UMCG, the Netherlands), University Groningen and the Northern Provinces of the Netherlands. Hanna van Loo is supported by a 2017 NARSAD Young Investigator Grant from the Brain & Behavior Research Foundation.

*The Netherlands Study of Depression and Anxiety (NESDA)*

The infrastructure for the NESDA study (www.nesda.nl) is funded through the Geestkracht program of the Netherlands Organisation for Health Research and Development (ZonMw, grant number 10-000-1002) and financial contributions by participating universities and mental health care organizations (VU University Medical Center, GGZ inGeest, Leiden University Medical Center, Leiden University, GGZ Rivierduinen, University Medical Center Groningen, University of Groningen, Lentis, GGZ Friesland, GGZ Drenthe, Rob Giel Onderzoekscentrum). This research was financially supported by BBMRI-NL, a Research Infrastructure financed by the Dutch government (NWO 184.021.007).

*Netherlands Twin Register (NTR)*

Funding was obtained from the Netherlands Organization for Scientific Research (NWO) and The Netherlands Organisation for Health Research and Development (ZonMW) grants 904-61-090, 985-10-002, 912-10-020, 904-61-193,480-04-004, 463-06-001, 451-04-034, 400-05-717, Addiction-31160008, 016-115-035, 481-08-011, 056-32-010, Middelgroot-911-09-032, OCW_NWO Gravity program –024.001.003, NWO-Groot 480-15-001/674, Center for Medical Systems Biology (CSMB, NWO Genomics), NBIC/BioAssist/RK(2008.024), Biobanking and Biomolecular Resources Research Infrastructure (BBMRI –NL, 184.021.007 and 184.033.111); Spinozapremie (NWO- 56-464-14192), KNAW Academy Professor Award (PAH/6635) and University Research Fellow grant (URF) to DIB; Amsterdam Public Health research institute (former EMGO+), Neuroscience Amsterdam research institute (former NCA); the European Science Foundation (ESF, EU/QLRT-2001-01254), the European Community's Seventh Framework Program (FP7- HEALTH-F4-2007-2013, grant 01413: ENGAGE and grant 602768: ACTION); the European Research Council (ERC Starting 284167, ERC Consolidator 771057, ERC Advanced 230374), Rutgers University Cell and DNA Repository (NIMH U24 MH068457-06), the National Institutes of Health (NIH, R01D0042157-01A1, R01MH58799-03, MH081802, DA018673, R01 DK092127-04, Grand Opportunity grants 1RC2 MH089951, and 1RC2 MH089995); the Avera Institute for Human Genetics, Sioux Falls, South Dakota (USA). Part of the genotyping and analyses were funded by the Genetic Association Information Network (GAIN) of the Foundation for the National Institutes of Health. Computing was supported by NWO through grant 2018/EW/00408559, BiG Grid, the Dutch e-Science Grid and SURFSARA.

*Nijmegen Biomedical Study (NBS)*

The Nijmegen Biomedical Study is a population-based survey conducted at the Department for Health Evidence and the Department of Laboratory Medicine of the Radboud university medical center. Principal investigators of the Nijmegen Biomedical Study are L.A.L.M. Kiemeney, A.L.M. Verbeek, D.W. Swinkels en B. Franke.

*Nutrition Questionnaires plus (NQplus)*

The NQplus study was core funded by ZonMw (ZonMw Grant 91110030).

*TRacking Adolescents’ Individual Lives Survey (TRAILS)*

Participating centers of the TRacking Adolescents' Individual Lives Survey (TRAILS) include the University Medical Center and University of Groningen, the University of Utrecht, the Radboud Medical Center Nijmegen, and the Parnassia Group, all in the Netherlands. TRAILS has been financially supported by various grants from the Netherlands Organization for Scientific Research NWO (Medical Research Council program grant GB-MW 940-38-011; ZonMW Brainpower grant 100-001-004; ZonMw Risk Behavior and Dependence grants 60-60600-97-118; ZonMw Culture and Health grant 261-98-710; Social Sciences Council medium-sized investment grants GB-MaGW 480-01-006 and GB-MaGW 480-07-001; Social Sciences Council project grants GB-MaGW 452-04-314 and GB-MaGW 452-06-004; NWO large-sized investment grant 175.010.2003.005; NWO Longitudinal Survey and Panel Funding 481-08-013 and 481-11-001; NWO Vici 016.130.002 and 453-16-007/2735; NWO Gravitation 024.001.003), the Dutch Ministry of Justice (WODC), the European Science Foundation (EuroSTRESS project FP-006), the European Research Council (ERC-2017-STG-757364 en ERC-CoG-2015-681466), Biobanking and Biomolecular Resources Research Infrastructure BBMRI-NL (CP 32), the Gratama foundation, the Jan Dekker foundation, the participating universities, and Accare Centre for Child and Adolescent Psychiatry. Statistical analyses are carried out on the Genetic Cluster Computer (http://www.geneticcluster.org), which is financially supported by the Netherlands Scientific Organization (NWO 480-05-003) along with a supplement from the Dutch Brain Foundation. We are grateful to everyone who participated in this research or worked on this project to make it possible. We are grateful to all adolescents, their parents and teachers who participated in this research and to everyone who worked on this project and made it possible.

# Genotyping, imputation and PCA in NTR

Genotyping was done on several platforms, namely Perlegen Affymetrix, Affymetrix 6.0, Affymetrix Axiom, Illumina 660, Illumina 1M, Illumina GSA. Part of the sample was whole genome sequenced in Genome of the Netherlands (GoNL) project ([Boomsma et al., 2014](#_ENREF_3)). Within each platform and on combined dataset samples and single nucleotide polymorphism (SNP) quality control (QC) was performed. Samples were removed if sex did not match the expected, heterozygosity F statistic was < -0.10 or > 0.10, call rate was < 0.90. SNPs were removed if minor allele frequency (MAF) was <0.01, Hardy-Weinberg Equilibrium (HWE) p-value < 1×10^-5^, call rate < 0.95, number of Mendel errors < 20. In addition AT/GC SNPs with MAF range between 0.4 and 0.5 were removed to avoid wrong strand alignment to the reference panel. When datasets were combined into one (except GoNL sequence data and only including 1 MZ twin per pair), the familial relationships between family members were checked to correspond to expected. The combined dataset was on positive strand and was based on build 37 (HG19). Familial relationships were checked separately in GoNL sequence data. Combined set was aligned to GoNL reference set and SNPs, which differed in allele frequency > 0.10, have mismatching alleles, location or strand were removed. Then data were cross-platform imputed using MaCH-Admix against GoNL reference set to predict the missing SNPs in each platform based on haplotypes estimated from Dutch reference panel ([Deelen et al., 2014](#_ENREF_7); [Fedko et al., 2015](#_ENREF_8); [Liu, Li, Wang, & Li, 2013](#_ENREF_10)). SNPs were removed from cross-platform imputed dataset if HWE p-value was < 1×10^-5^, Mendel error rate was more than mean + 3SD, R^2^ imputation quality metric was < 0.90, if p-value for association with a platform was < 1×10^-5^. GoNL sequence data were added after imputation. Ethnic outliers (non-Dutch ancestry) were defined based on Principal Components Analysis (PCA) by projecting 10 PCs from 1000G reference set populations on NTR cross-platform imputed data using EIGENSTRAT ([Price et al., 2006](#_ENREF_18)). Individuals with PC values located outside of the range of European and/or British populations were defined as outliers. Upon exclusion of outliers, 10 PCs were recomputed for NTR cross-platform imputed data to capture the variation within the Netherlands ([Abdellaoui et al., 2013](#_ENREF_1)).

# Polygenic Risk Score (PRS) analysis in NTR

We computed PRS based on cross-platform imputed data (excluding ethnic outliers) using LDpred software ([Vilhjálmsson et al., 2015](#_ENREF_26)). It allows to include all SNPs in PRS calculation by modeling linkage disequilibrium (LD) structure between them. LD structure was estimated based on the same NTR cross-platform imputed data, except that only unrelated individuals were included (N=9,941). The total number of SNPs was 1,302,481. LDpred uses all SNPs for PRS calculation, however, models the assumed causal fraction of SNPs. We assumed causal fraction of 0.3 and used summary statistics from the largest MDD GWAS meta-analysis to date, which was re-run excluding NTR and 23andMe data ([Wray et al., 2018](#_ENREF_28)). We predicted MDD case control status in NTR data using generalized estimating equation (GEE) with binomial link function, implemented as a package in R, to account for familial relatedness between NTR individuals, with age, sex, 10 PCs and 6 genotyping chips as fixed covariates ([Minică, Dolan, Kampert, Boomsma, & Vink, 2015](#_ENREF_13); [R Core Team, 2016](#_ENREF_20)).

# Comparison of BIONIC respondents and other cohort participants for personality traits and/or other depression related phenotypes

We compared BIONIC respondents against other individuals in NTR to evaluate whether there is a difference between the two groups with respect to 1) neuroticism, assessed by NEO Five-Factor Inventory (NEO-FFI) ([Costa Jr & McCrae, 1989](#_ENREF_6); [McCrae & Costa Jr, 2007](#_ENREF_12)); 2) anxiety, assessed through the Anxious-Depressed scale of Adult Self Report (ASR) questionnaire ([Achenbach & Rescorla, 2003](#_ENREF_2)); 3) depression, assessed through Hospital Anxiety and Depression Scale (HADS) ([Zigmond & Snaith, 1983](#_ENREF_29)) and 4) answer to the question “Ever been in contact with counselling professionals for problems unrelated to physical health?”. When measurements were available in multiple surveys, we took the measurement that was closer to LIDAS assessment with the corresponding age. In addition to visual examination of the variable distributions in two groups, we compared means using independent t-test for neuroticism (normally distributed) and Wilcoxon rank sum test for anxiety and depression (skewed distribution). We also compared if proportions of BIONIC respondents differs between those who answered to the question about seeking help for not physical complaints, namely “No”, ”Yes, in the past, not anymore”, ”Yes, now”. We randomly selected 1 person per family to ensure that individuals are unrelated.

BIONIC respondents and other NTR participants did not differ significantly in relation to ASR anxious-depressed scale tested with Wilcoxon rank sum test (N_BIONIC_ = 3,505; N_OTHER_ = 5,406; Median_BIONIC_=3.0; Median_OTHER_=3.0; p=0.11; Supplementary Figure 1a) and neuroticism tests with independent t-test (N_BIONIC_=3,450; N_OTHER_=5,478; Mean_BIOINIC_=30.0, Mean_OTHER_=30.5,p=0.013, Supplementary Figure 1b). Significant difference was detected for HADS depression (N_BIONIC_ = 2,095; N_OTHER_ = 3,196; Median_BIONIC_=2.0; Median_OTHER_=2.0; p= 6.15×10^-6^; Supplementary Figure 1c) and proportions of BIONIC participants across three answers to the question about contact with counselling professionals (Prop1(“no”)=0.37, Prop2 (“yes, in the past not anymore”)=0.42, Prop3(“yes”)=0.45, p=7.59×10^-5^). We also found significant differences, when answers to three questions indicated above were compared across BIONIC and non BIONIC participants (1. Answer “No” prop1=0.67, prop2=0.72, χ^2^=17.06, df=1, p=3.62×10^-5^; 2. Answer “yes, in the past not anymore” prop1=0.26, prop2=0.23, χ^2^=8.18, df=1, p=0.004; 3. Answer “Yes” prop1=0.07, prop2=0.05, χ^2^=7.57, df=1, p=0.006; Supplementary Figure 1d). We applied Bonferroni correction taking into account number of proportion comparisons, 0.05/4=0.0125. Although statistically significant, the differences appear to be small and are not likely to be relevant.

Supplementary Figure 1. Comparison of BIONIC respondents against other individuals to evaluate whether there is a difference between two groups with respect to neuroticism, anxiety, depression, contact with counselling professionals for problems unrelated to physical health.

**
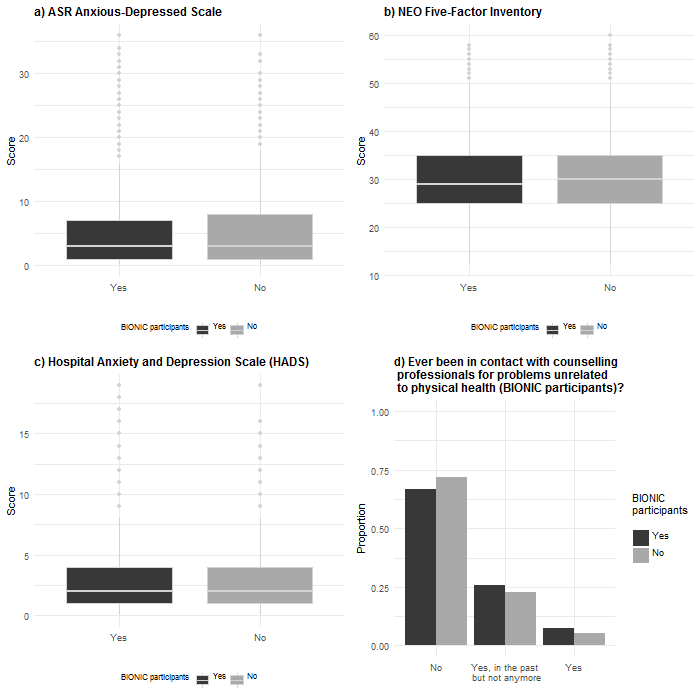
**

# Cut-off for low, medium and high education levels of BIONIC cohorts*

For all cohorts except Lifelines we categorized level of education as low (primary school, special education, lower general secondary [VMBO/vocational, LTS]), medium (extended primary education [ULO], lower general secondary [MULO, MAVO, VMBO theoretical], 2-4 years of intermediate vocational education [MBO], upper general secondary school [HAVO, HBS, VWO, gymnasium]), and high (higher vocational education [HBO], university) irrespective of whether diploma or certificate received, with exception of the high education group. If no diploma or certificate was received in that group, a participant would be included in the medium education group.

For the Lifelines cohort, the question about education was slightly different (“What is your highest level of completed education?”) and already assumed diploma or certificate being received and data were categorized taking it into account. Namely low education level included no education (primary school or primary education not completed) or primary education (primary education, specialized primary education); medium education level included lower or preparatory vocational education (such as LTS, LEAO, LHNO, VMBO), lower general secondary education (such as MAVO, (M)ULO, MBO-short, VMBO-t), lower vocational education (such as MBO-long, MTS, MEAO, BOL, BBL, INAS); high education level included higher general secondary education and preparatory academic education (such as HAVO, VWO, Atheneum, Gymnasium, HBS, MMS), higher vocational education (such as HBO, HTS, HEAO, candidates for academic education, bachelor's) and academic education (university).

* Dutch education system abbreviations (in Dutch):

LTS – lagere technische school

LEAO – lager economisch en administratief onderwijs

LHNO – lager huishoud- en nijverheidsonderwijs

(M)ULO – (meer) uitgebreid lager onderwijs

MAVO – middelbaar algemeen voortgezet onderwijs

VMBO – voorbereidend middelbaar beroepsonderwijs

MBO – middelbaar beroepsonderwijs

MTS – middelbare technische school

MEAO – middelbare economisch en administratief onderwijs

MMS – middelbare meisjesschool

BOL – beroeps opleidende leerweg

BBL – beroeps begeleidende leerweg

INAS – inrichtingsassistente

HAVO – hoger algemeen voortgezet onderwijs

HBS – hogere burgerschool

HTS – hogere technische school

HEAO – hoger economisch en administratief onderwijs

VWO – voorbereidend wetenschappelijk onderwijs

HBO – hoger beroepsonderwijs

# Genetic correlation analyses

Genetic correlations were estimated in two ways. First, a bivariate variance component analysis (VCA) was performed analyzing LIDAS and CIDI MDD data in the Mendel software package for pedigree analysis (Lange et al., 2013). The overlap between MDD assessed with the LIDAS (*N* = 18,103) and clinical MDD assessed with the CIDI (*N* = 6,507) ([Mbarek et al., 2017](#_ENREF_11)) comprised 1,682 individuals with pedigree, age and sex data present, and after the removal of 59 ancestry outliers. Clinical MDD status was assessed by trained research staff according to the CIDI based on DSM-IV criteria ([Penninx et al., 2008](#_ENREF_16); [Sullivan et al., 2009](#_ENREF_22)). The overlapping sample contained 131 MZ twin pairs, 47 DZ twin pairs, and 1,179 familial pedigrees in total. Supplementary Table 2 contains an overview of MDD status in participants who were present in both datasets. The large majority (91.4%) of participants were concordant in their lifetime MDD status, and 131 participants who previously were controls according to the CIDI had become cases. There were 13 cases who were no longer assigned case status. In the bivariate variance components analysis, sex and standardized age were added as fixed effects, and a shared household variable was defined for twins. The genetic correlation between LIDAS and CIDI MDD data was calculated from the Mendel output by dividing the estimated genetic covariance *(cov)* by the product of the square root of the additive genetic variance components (*var1* and *var2*); $\frac{cov}{\sqrt{\left( var1 \right)\times(var2)}}$. The covariance of the additive genetic effects was 0.0349 (*SE* = 0.0028). Additive genetic variance components were 0.0522 (*SE* = 0.0046) and 0.0473 (*SE* = 0.0020) for LIDAS and CIDI MDD respectively. The genetic correlation equaled 0.70.

Second, we attempted to estimate the genetic correlation using the Linkage Disequilibrium Score (LDSC) regression ([Bulik-Sullivan et al., 2015](#_ENREF_1)) framework. To obtain the required summary statistics for LDSC regression we ran two separate GWASs for MDD assessed with LIDAS (*N* = 6,254) and CIDI (*N* = 5,970) in PLINK version 1.07 ([Purcell et al., 2007](#_ENREF_6)), using sex, age, 6 genotyping platforms and 10 principal components as covariates. These sample sizes apply after exclusion of 424 ancestral outliers from the LIDAS sample, and 188 from the CIDI sample. The estimate of SNP-heritability in the LIDAS MDD dataset was too low (SNP-*h*² = -0.0482, *SE* = 0.0816) to allow estimating the genetic correlation. We conclude that the sample is currently of inadequate size to estimate a genetic correlation based on the SNP data.

# LIDAS questionnaire

**BIONIC – Survey about mood and health**

| Collaboration between <study name> and BBMRI-NL  Welcome to the survey of the BIONIC research. Please complete all the questions. Please select the answer that is most appropriate for you. Questions that are not applicable to you, will be skipped automatically.  The completion of the questionnaire will take 5-10 minutes. If you are not able to complete the questionnaire at once, you can complete it in multiple stages. The results will be saved, and you can continue the questionnaire at a later time point.  Do you have any questions? Please check <this page> for frequently asked questions and answers. If your question is not listed there, you can contact the research assistant (see information letter for contact details).  Thank you for your participation. |
| --- |

| **A – Deelname** |
| --- |

We would like to ask you to read the information below carefully and tick the box. After that you get access to the questionnaire.

1. □ By crossing this box I declare that:

- I read the information letter for participants. I understood the information.
- I give permission to add the answers to this questionnaire to previously collected data by <study name>.
- I know that my participation is voluntary. I know that I can decide to stop my participation at any time.

| **B – Biografische vragen en leefstijl** |
| --- |

1. I am a □_0_ Male □_1_ Female
2. What is your age? year

**3.** What is the highest level of education that you followed? *(in brackets Dutch names)*

□_1_ Primary school

□_2_ Special education

□_3_ Lower general secondary (vmbo/vocational stream, lts)

□_4_ Extended primary education (ulo)

□_5_ Lower general secondary (mulo, mavo, vmbo theoretical)

□_6_ 2-4 years of intermediate vocational education (mbo)

□_7_ Upper general secondary school (havo, hbs, vwo, gymnasium)

□_8_ Higher vocational education (hbo)

□_9_ University

□_10_ Other, namely:

**4.** Did you complete this education with a diploma or certificate? □_1_ Yes □_0_ No

**5.** Do you smoke? □_0_ No, I have never smoked □_1_ I do not smoke at the moment, but I have smoked in the past □_2_ Yes

**6.** Do you at least once per week engage in physical activities in your leisure time that cause sweating?

□_0_ Nee Yes, namely □_1_ once per week □_2_ twice per week □_3_ three times per week □_4_ four times per week or more

| **C – Stemming en gezondheid** |
| --- |

The next questions are about periods of sadness and depression in your life.

**7.** Have you ever had a time in your life when you felt sad, empty or depressed for two weeks or longer**?**

□_1_ Yes □_0_ No ► **go to question 9**

**8.** For the next question, think about a period of two weeks in your life when these feelings were worst. During this period of two

weeks, how often did you feel this way?

□_1_ Almost every day, most of the day **► go to question 11**

□_0_ Less often

**9.** Have you ever had a time in your life lasting two weeks or more when you lost interest in most things like hobbies, work, or activities that usually give you pleasure?

□_1_ Yes □_0_ No ► **go to question 35**

**10.** For the next question, think about the period of two weeks in your life when your loss of interest was worst. During this

period of two weeks, how often did you feel this way?

□_1_ Almost every day, most of the day ► **go to question 13**

□_0_ Less often ► **go to question 35**

**11.** During this period that you felt sad, empty or depressed, did you ever have a period lasting two weeks or more when you lost interest in most things like hobbies, work, or activities that usually give you pleasure?

□_1_ Yes □_0_ No ► **go to question 13**

**12.** For the next question, think about the period of two weeks in your life when your loss of interest were worst. During this

period of two weeks, how often did you feel this way?

□_1_ Almost every day, most of the day □_0_ Less often

**People who have periods in which they feel sad, empty or depressed or lose interest, often have other problems at the**

**same time. During this period of at least two weeks when you felt sad, empty or depressed or lost interest in things:**

**13.** … did you lack energy or feel tired more than usual?

□_1_ Yes □_0_ No

**14.** … did you have less appetite than usual almost every day?

□_1_ Yes □_0_ No

**15.** … did you lose weight without trying to, as much as a kilo a week for several weeks?

□_1_ Yes □_0_ No ► **go to question 17**

**16.** About how much weight did you lose in these weeks? kg

**During this period of at least two weeks when you felt sad, empty or depressed or lost interest in things:**

**17.** … did you have a much larger appetite than usual almost every day for two weeks or more?

□_2_ Yes □_1_ Yes, only because of pregnancy or a growth spurt □_0_ No

**18.** … did your eating increase so much that you gained weight, as much as a kilo a week for several weeks?

□_1_ Yes □_0_ No ► **go to question 20**

**19.** About how much weight did you gain in these weeks? kg

**During this period of at least two weeks when you felt sad, empty or depressed or lost interest in things:**

**20.** …. did you have trouble sleeping almost every night, either trouble falling asleep, waking in the middle of the night, or

waking up too early?

□_1_ Yes □_0_ No ► **go to question 22**

**21.** …. did you wake up at least two hours before you wanted to, every day for at least two weeks?

□_1_ Yes □_0_ No

**22.** …. were you sleeping too much almost every day?

□_1_ Yes □_0_ No

**During this period of at least two weeks when you felt sad, empty or depressed or lost interest in things:**

**23.** …. did you talk or move more slowly than is normal for you almost every day, in a way that other people have noticed?

□2 Yes, I talked or moved more slowly and other people did notice

□1 Yes, I talked or moved more slowly but other people did not notice

□0 No

**24**. .… did you have to be moving all the time, that is, you couldn’t sit still and paced up and down or couldn’t keep your hands still when sitting, in a way that other people noticed?

□2 Yes, I had to be moving all the time and other people did notice

□1 Yes, I had to be moving all the time but other people did not notice

□0 No

**25.** During this period of two weeks, did you have a lot more trouble concentrating than usual?

□_1_ Yes □_0_ No

**26.** During this period, were you able to make up your mind about things you ordinarily had no trouble deciding about?

□_1_ Yes □_0_ No

**27.** People sometimes feel down on themselves, no good, or worthless. During this period of two weeks, did you feel guilty or

worthless?

□_1_ Yes □_0_ No

**28.** During this period of two weeks, did you think a lot about death – either your own, someone else’s, or death in general?

□_1_ Yes □_0_ No

**For the next question, please think about the periods of at least two weeks in your life when you when you felt sad, empty or depressed or lost interest in things.**

**29.** Did any period lasting two weeks or longer seriously interfere with your ability to do your job, take care of your house or family, or

take care of yourself?

□_1_ Yes □_0_ No

**30.** About how long did the longest of periods like this last? *You may give an estimate.*

weeks

**31.** How many periods like this did you have in your life?

□_1_ 1 □_2_ 2 or more

**32.** Did you have a period like this in the last 12 months?

□_1_ Yes □_0_ No

**33.** About how old were you the first time you had a period like this? (whether or not you received any help for it)

years

**34.** Did you ever tell a professional about these problems (for example a medical doctor, psychologist, social worker,

nurse, or other helping professional)?

□_1_ Yes □_0_ No

**35.** Have you ever had contact with a social service organization for problems that you had, that were not related to your physical

health? (for example a mental health institute, psychologist, or social work)

□_2_ Yes □_1_ Yes in the past, not anymore □_0_ No

**36.** Please tick the disorders that you have ever been diagnosed with by a professional or medical doctor. *You can tick more than*

*one answer.*

Ever diagnosed by a professional/medical doctor:

□_a_ Depression □_h_ Post traumatic stress disorder

□_b_ Bipolar disorder (manic depression) □_i_ Phobia

□_c_ Schizophrenia or psychosis □_j_ ADD/ADHD

□_d_ Eating disorder □_k_ Personality disorder

□_e_ Anxiety disorder □_l_ Alcohol addiction

□_f_ Panic disorder □_m_ Drug addiction

□_g_ Obsessive compulsive disorder □_n_ Other, namely:

□_o_ None of these disorders ► **go to question 38**

**37.** Please tick the disorders that you have ever received treatment for by a professional or medical doctor. *You can tick more*

*than one answer.*

Ever received treatment by a professional/medical doctor:

□_a_ Depression □_i_ Phobia

□_b_ Bipolar disorder (manic depression) □_j_ ADD/ADHD

□_c_ Schizophrenia or psychosis □_k_ Personality disorder

□_d_ Eating disorder □_l_ Alcohol addiction

□_e_ Anxiety disorder □_m_ Drug addiction

□_f_ Panic disorder □_n_ Other, namely:

□_g_ Obsessive compulsive disorder □_o_ None of these disorders

□_h_ Posttraumatic stress disorder

**38.** Did you ever have the following treatments? *You can tick multiple answers.*

□a Antidepressants

□b Psychotherapy (for example cognitive behavioral therapy, interpersonal therapy or other consults with a psychologist or

psychotherapist)

□c Online help program or e-health intervention

□d Running therapy or physical activity

□e Light therapy

□f Hospitalization in psychiatric hospital

□g Electroconvulsive therapy (ECT)

□h Other, namely:

□i None of these treatments

**39.** What is your length in cm? *Please round your answer to the nearest whole number.*

cm

**40.** What is your weight in kg? *Please round your answer to the nearest whole number.*

kg

**41. This is the end of the questionnaire. Do you have any comments?**

□ No

□ Yes, namely:

**^41a.^**

**Thank you very much for taking part in this survey!**

You can now close the questionnaire.

Supplementary Figure 2. Pooled estimates of **current MDD** prevalence of participants with LIDAS data, stratified by a) sex, b) age, c) education, d) smoking, e) physical activity and f) obesity

a)


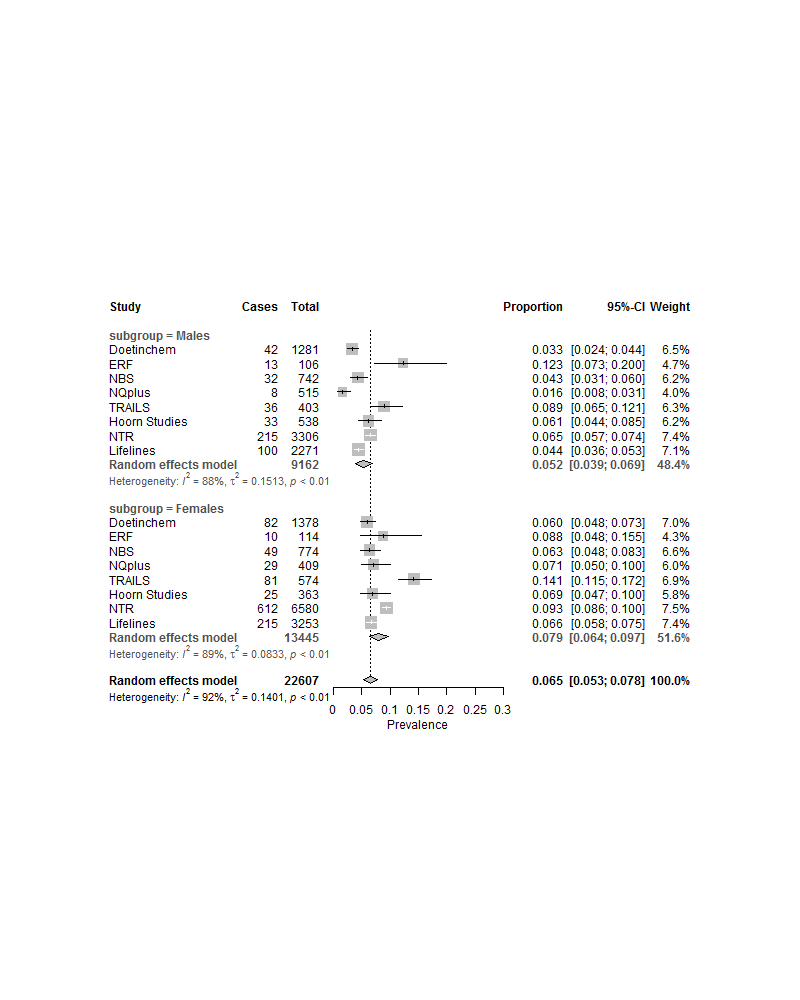


b)


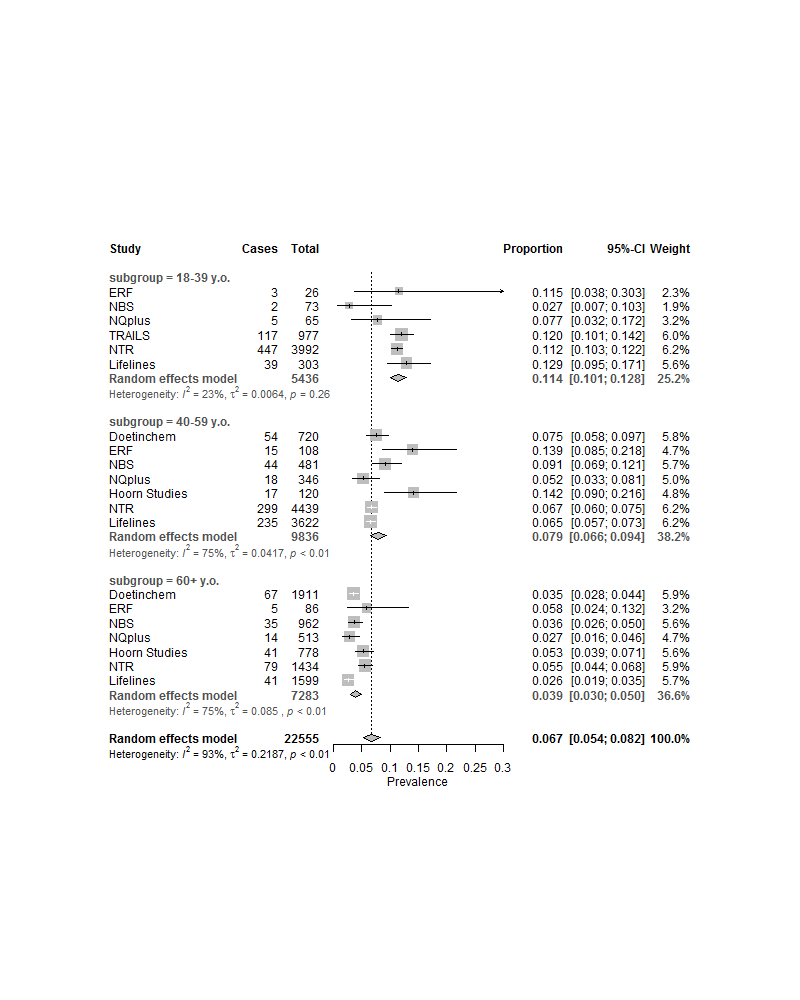


c)


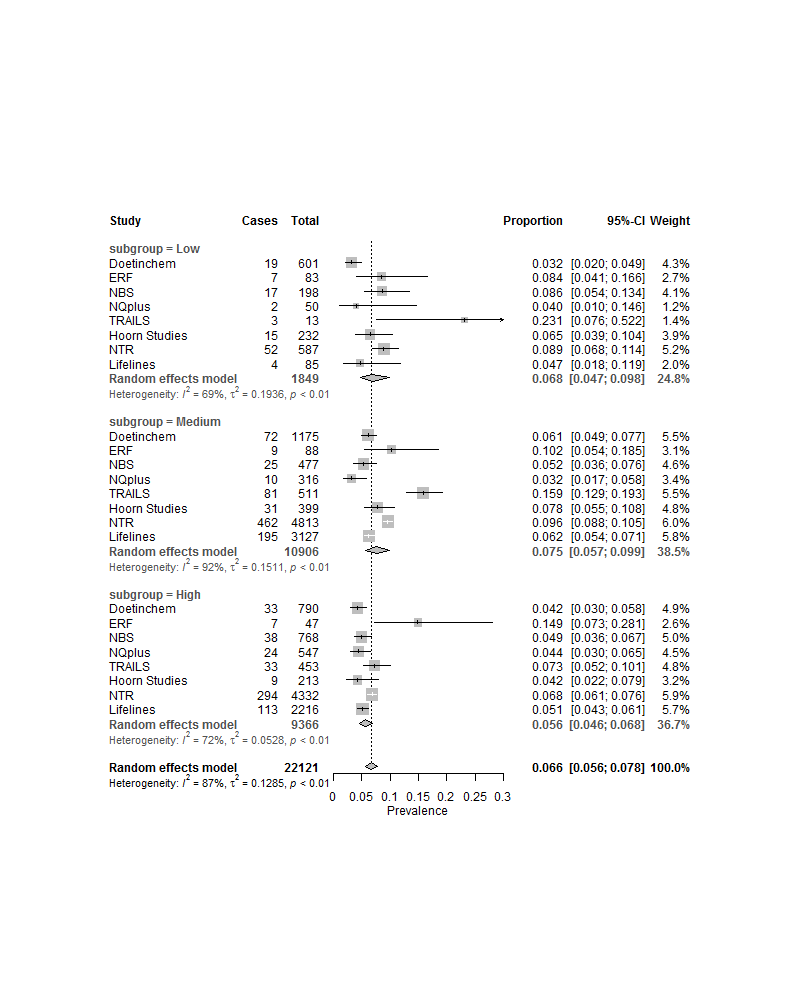


d)


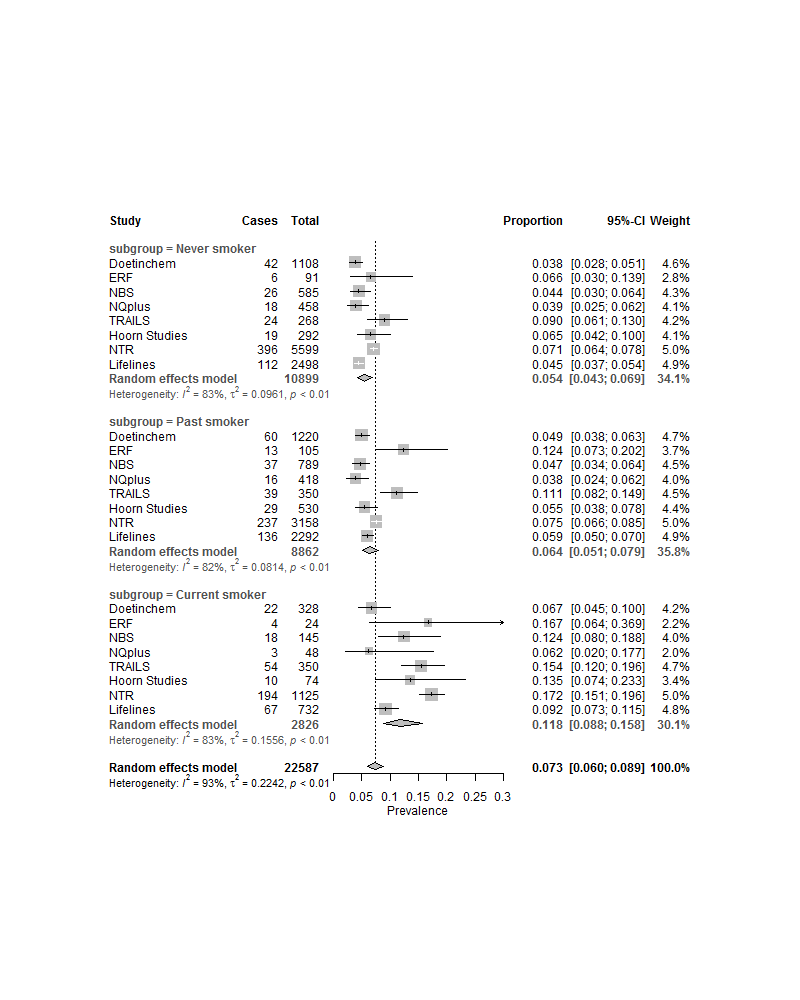


e)


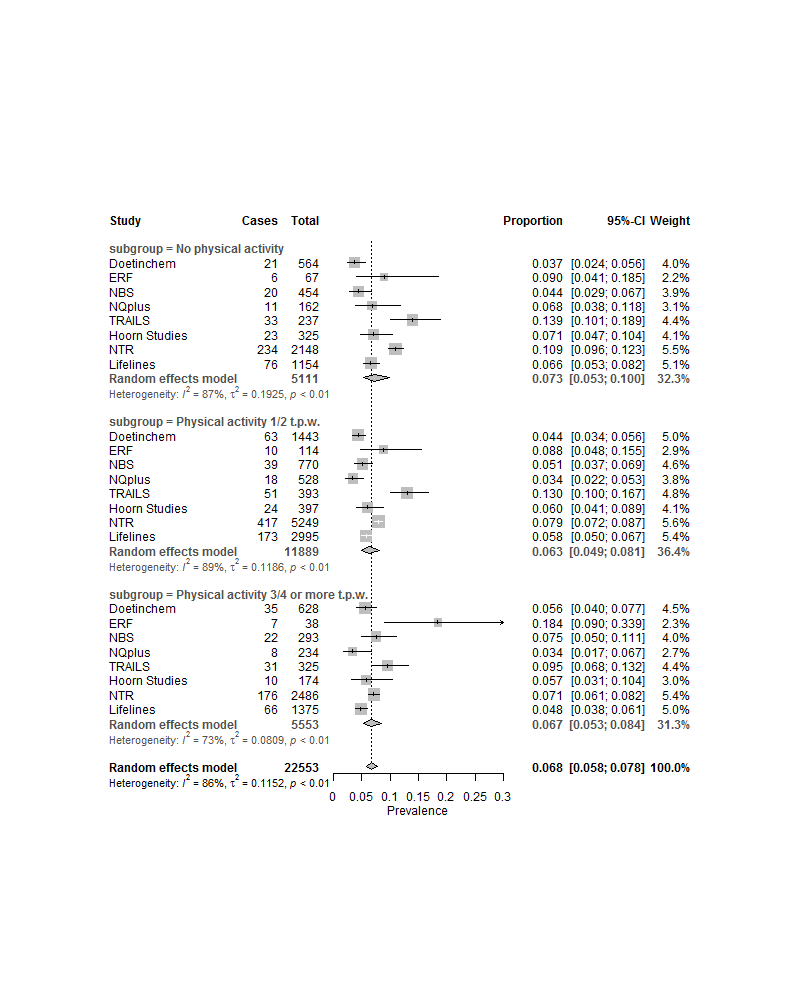


f)


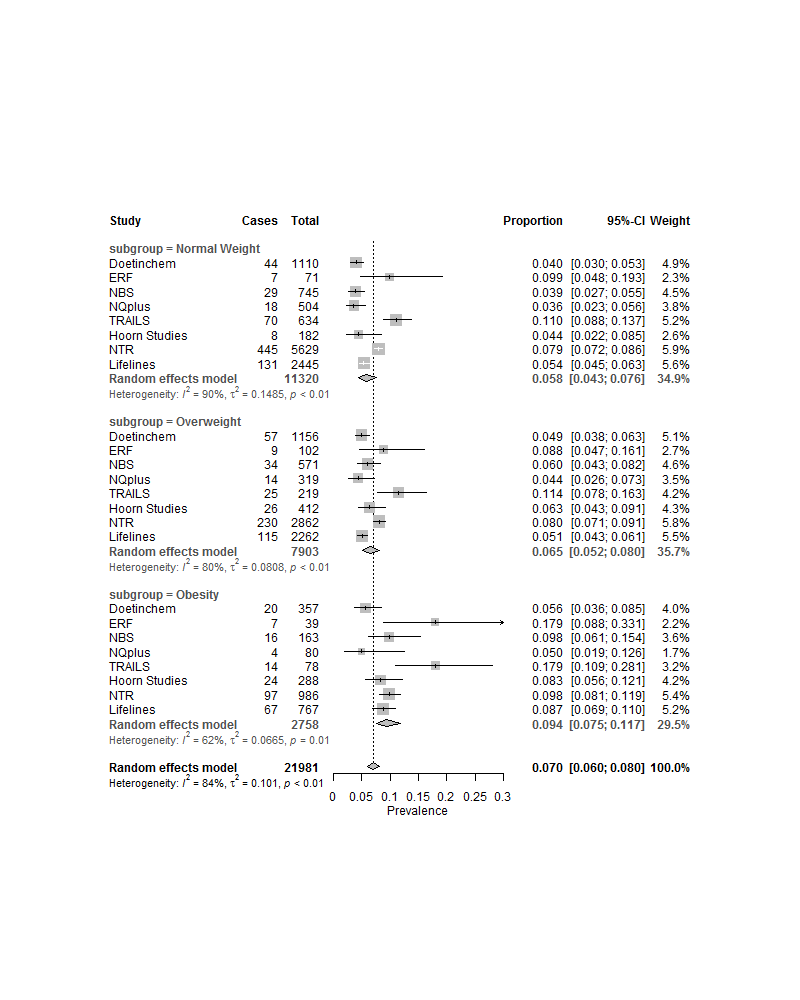


Supplementary Figure 3. Pooled estimates of **lifetime MDD** prevalence of participants with LIDAS data, stratified by a) sex, b) age, c) education, d) smoking, e) physical activity and f) obesity

a)


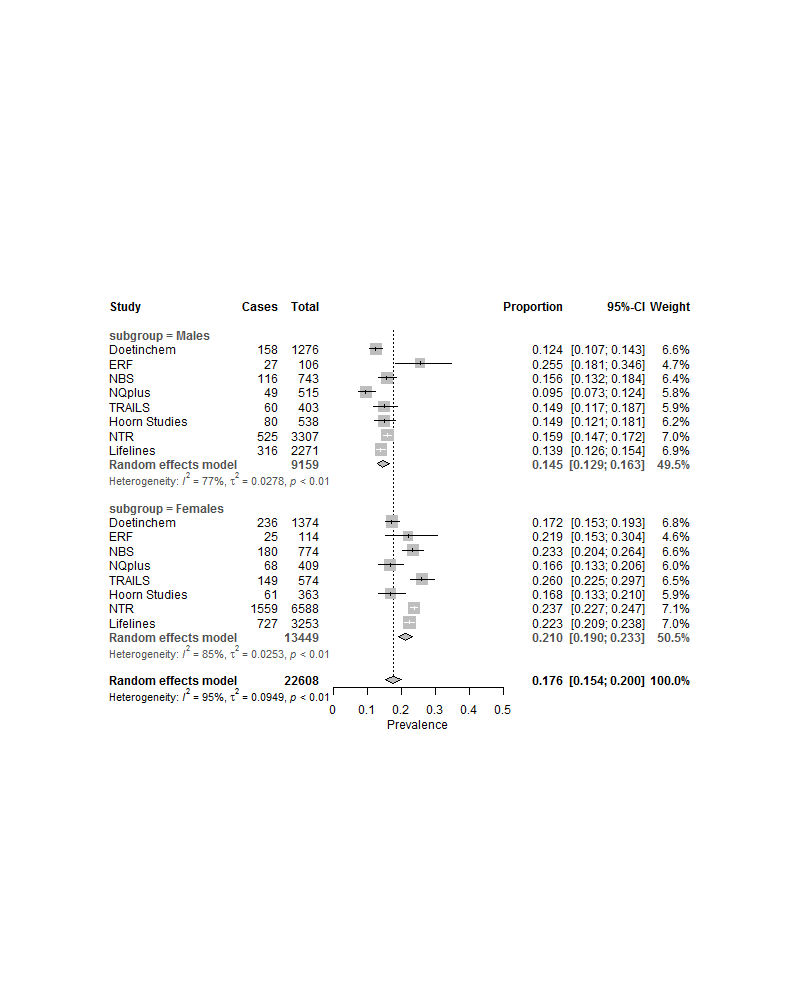


b)
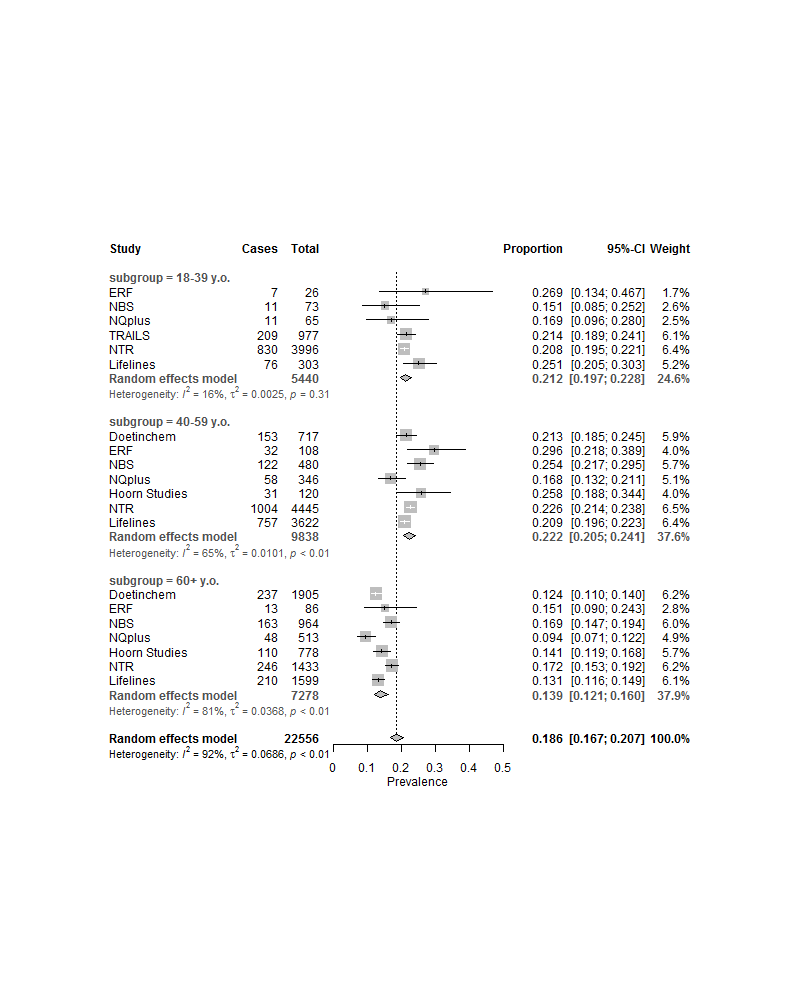


c)


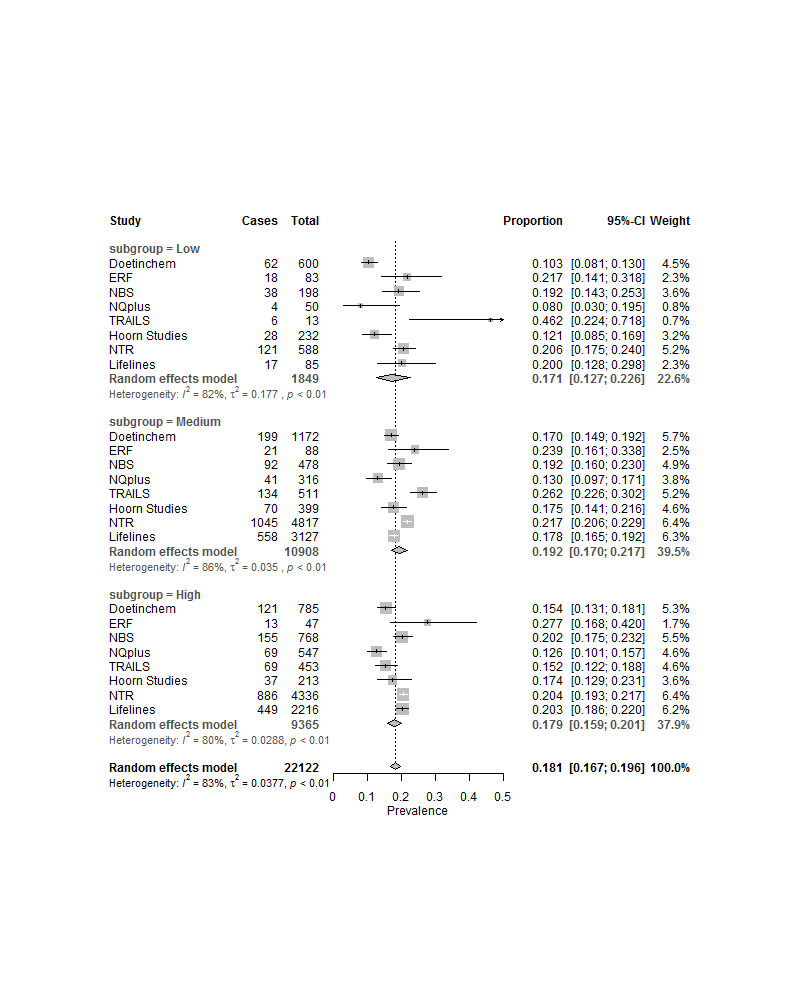


d)


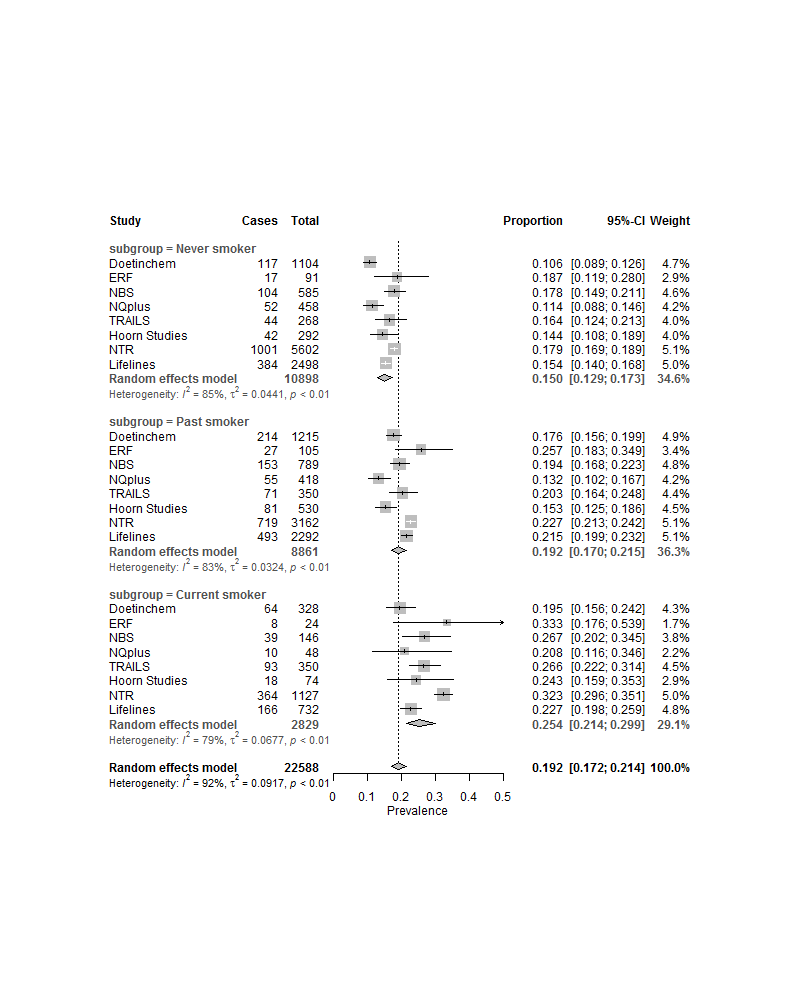


e)


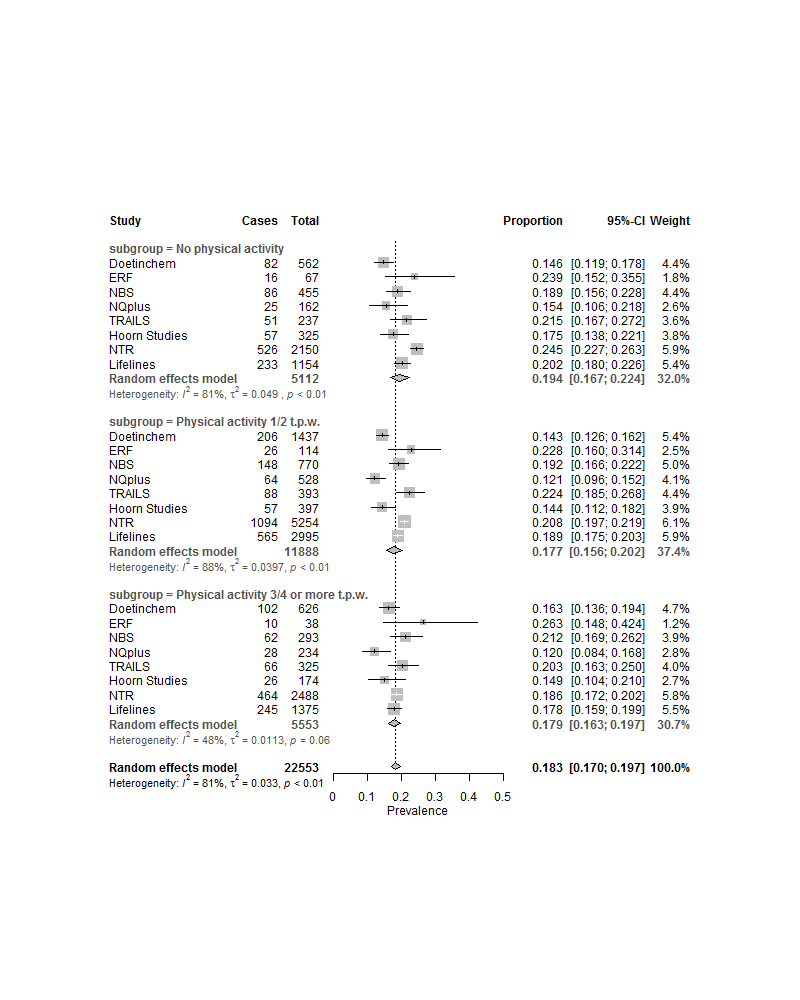


f)


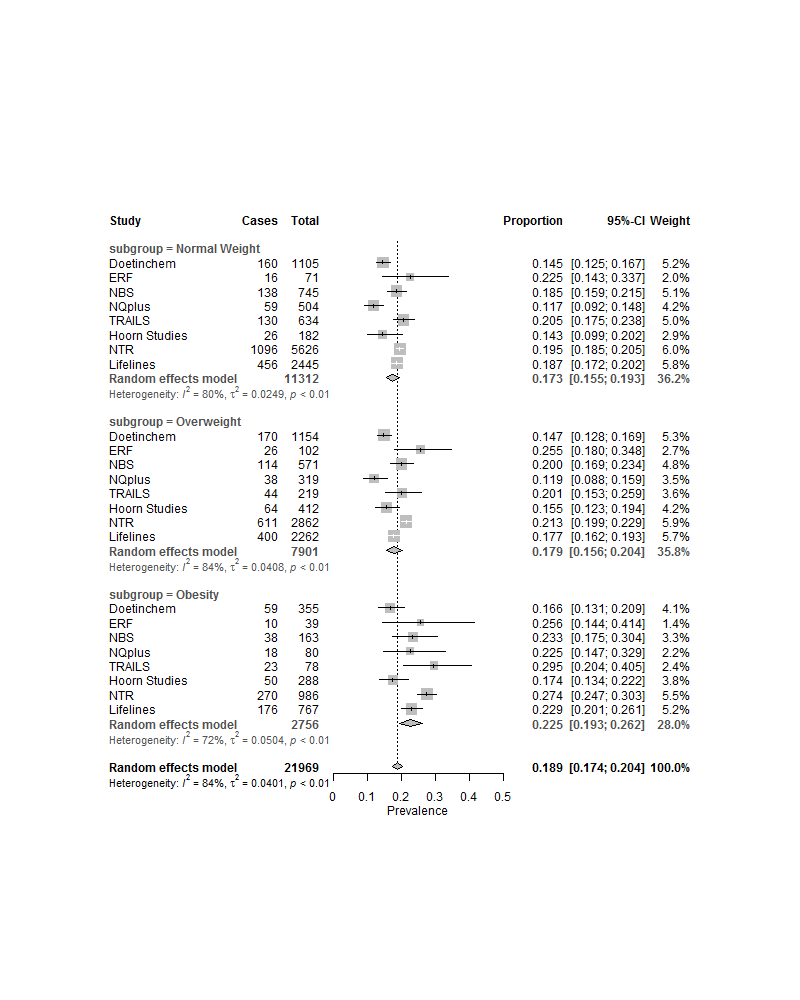


Supplementary Figure 4. Forest plots of the leave-one-out analysis with pooled estimates of a) current and b) lifetime MDD prevalence

| a) | 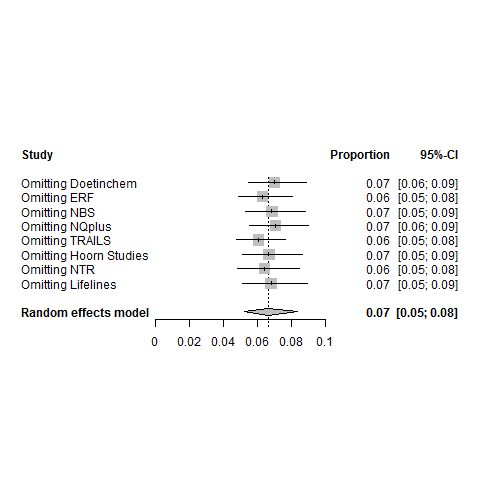 |
| --- | --- |
| b) | 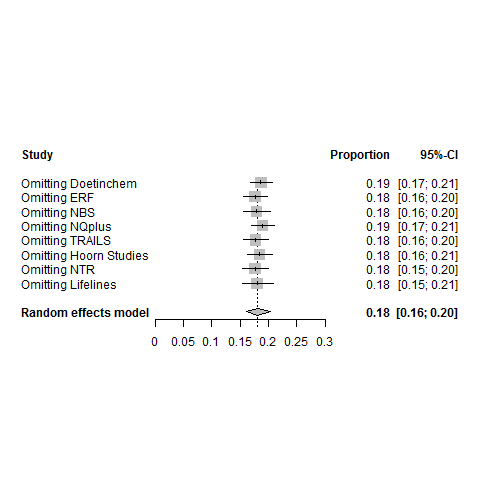 |

# Supplementary Table 1. Estimates of fixed effects of age and sex and variance components (with standard errors): A represents additive genetic variance, C represents environmental variance common to twins from the same household and E represents unshared environmental variance.

|  | Lifetime MDD | Current MDD | Height (cm) | Weight (kg) |
| --- | --- | --- | --- | --- |
| Age (SE) | -0.0001 (0.0002) | -0.002 (0.0002) | -0.06 (0.003) | 0.20 (0.006) |
| Sex (SE), males | -0.05 (0.004) | -0.02 (0.003) | 6.58 (0.05) | 6.06 (0.10) |
| Sex (SE), females | 0.05 (0.004) | 0.02 (0.003) | -6.58 (0.05) | -6.06 (0.10) |
| A (SE) | 0.05 (0.005) | 0.03 (0.003) | 36.26 (0.88) | 89.99 (3.52) |
| C (SE) | 0.002 (0.005) | 0.00 (-) | 3.08 (0.69) | 41.86 (3.15) |
| E (SE) | 0.12 (0.004) | 0.06 (0.002) | 5.23 (0.21) | 32.29 (1.37) |
| A, % | 0.30 | 0.34 | 0.81 | 0.55 |
| C, % | 0.01 | 0 | 0.07 | 0.26 |
| E, % | 0.69 | 0.66 | 0.12 | 0.20 |

# Supplementary Table 2. The concordance between MDD statuses assessed with LIDAS and CIDI instruments.

|  |  | Clinical MDD | |
| --- | --- | --- | --- |
|  |  | Cases | Controls |
| LIDAS-MDD | Cases | 55 | 131 |
|  | Controls | 13 | 1483 |

# **References:**

Abdellaoui, A., Hottenga, J.-J., de Knijff, P., Nivard, M. G., Xiao, X., Scheet, P., . . . Boomsma, D. I. (2013). Population structure, migration, and diversifying selection in the Netherlands. *European Journal of Human Genetics, 21*(11), 1277–1285.

Achenbach, T., & Rescorla, L. (2003). Manual for the ASEBA Adult Forms & Profiles. *Burlington, VT: University of Vermont, Research Center for Children, Youth & Families*.

Boomsma, D. I., Wijmenga, C., Slagboom, E. P., Swertz, M. A., Karssen, L. C., Abdellaoui, A., . . . van Duijn, C. M. (2014). The Genome of the Netherlands: design, and project goals. *European Journal of Human Genetics, 22*(2), 221-227.

Brouwer-Brolsma, E. M., Van Lee, L., Streppel, M. T., Sluik, D., Van De Wiel, A. M., De Vries, J. H., . . . Feskens, E. J. (2018). Nutrition questionnaires plus (nqplus) study, a prospective study on dietary determinants and cardiometabolic health in dutch adults. *BMJ open, 8*(7), e020228.

Bulik-Sullivan, B., Finucane, H. K., Anttila, V., Gusev, A., Day, F. R., Loh, P.-R., . . . Neale, B. M. (2015). An atlas of genetic correlations across human diseases and traits. *Nature genetics, 47*, 1236–1241.

Costa Jr, P., & McCrae, R. (1989). The NEO-PI/NEO-FFl manual supplement. *Odessa, FL: Psychological Assessment Resources*.

Deelen, P., Menelaou, A., van Leeuwen, E. M., Kanterakis, A., van Dijk, F., Medina-Gomez, C., . . . Swertz, M. A. (2014). Improved imputation quality of low-frequency and rare variants in European samples using the ‘Genome of The Netherlands’. *European Journal of Human Genetics, 22*, 1321–1326.

Fedko, I. O., Hottenga, J.-J., Medina-Gomez, C., Pappa, I., van Beijsterveldt, C. E., Ehli, E. A., . . . Boomsma, D. I. (2015). Estimation of Genetic Relationships Between Individuals Across Cohorts and Platforms: Application to Childhood Height. *Behavior genetics, 45*(5), 514-528.

Galesloot, T. E., Vermeulen, S. H., Swinkels, D. W., de Vegt, F., Franke, B., den Heijer, M., . . . Kiemeney, L. A. (2017). Cohort Profile: The Nijmegen Biomedical Study (NBS). *International journal of epidemiology, 46*(4), 1099-1100j.

Liu, E. Y., Li, M., Wang, W., & Li, Y. (2013). MaCH‐Admix: Genotype Imputation for Admixed Populations. *Genetic epidemiology, 37*(1), 25-37.

Mbarek, H., Milaneschi, Y., Hottenga, J.-J., Ligthart, L., de Geus, E. J., Ehli, E. A., . . . Boomsma, D. I. (2017). Genome-Wide Significance for PCLO as a Gene for Major Depressive Disorder. *Twin Research and Human Genetics*, 267-270.

McCrae, R. R., & Costa Jr, P. T. (2007). Brief versions of the NEO-PI-3. *Journal of individual differences, 28*(3), 116-128.

Minică, C. C., Dolan, C. V., Kampert, M. M., Boomsma, D. I., & Vink, J. M. (2015). Sandwich corrected standard errors in family-based genome-wide association studies. *European Journal of Human Genetics, 23*(3), 388-394.

Oldehinkel, A. J., Rosmalen, J. G., Buitelaar, J. K., Hoek, H. W., Ormel, J., Raven, D., . . . Hartman, C. A. (2015). Cohort profile update: the tracking adolescents’ individual lives survey (TRAILS). *International journal of epidemiology, 44*(1), 76-76n.

Pardo, L. M., MacKay, I., Oostra, B., van Duijn, C. M., & Aulchenko, Y. S. (2005). The effect of genetic drift in a young genetically isolated population. *Annals of human genetics, 69*(3), 288-295.

Penninx, B. W., Beekman, A. T., Smit, J. H., Zitman, F. G., Nolen, W. A., Spinhoven, P., . . . Van Dyck, R. (2008). The Netherlands Study of Depression and Anxiety (NESDA): rationale, objectives and methods. *International journal of methods in psychiatric research, 17*(3), 121-140.

Picavet, H. S. J., Blokstra, A., Spijkerman, A. M., & Verschuren, W. M. (2017). Cohort Profile Update: The Doetinchem Cohort Study 1987–2017: lifestyle, health and chronic diseases in a life course and ageing perspective. *International journal of epidemiology, 46*(6), 1751-1751g.

Price, A. L., Patterson, N. J., Plenge, R. M., Weinblatt, M. E., Shadick, N. A., & Reich, D. (2006). Principal components analysis corrects for stratification in genome-wide association studies. *Nature genetics, 38*(8), 904-909.

Purcell, S., Neale, B., Todd-Brown, K., Thomas, L., Ferreira, M. A., Bender, D., . . . Daly, M. J. (2007). PLINK: a tool set for whole-genome association and population-based linkage analyses. *The American Journal of Human Genetics, 81*(3), 559-575.

R Core Team. (2016). R: A language and environment for statistical computing. R Foundation for Statistical Computing. *Vienna, Austria. URL* [*https://www.R-project.org/*](https://www.R-project.org/)*.*

Rutters, F., Nijpels, G., Elders, P., Stehouwer, C. D., van der Heijden, A. A., Groeneveld, L., . . . Beulens, J. W. (2017). Cohort profile: the hoorn studies. *International journal of epidemiology, 47*(2), 396-396j.

Sullivan, P. F., de Geus, E. J., Willemsen, G., James, M. R., Smit, J. H., Zandbelt, T., . . . Penninx, B. W. (2009). Genome-wide association for major depressive disorder: a possible role for the presynaptic protein piccolo. *Molecular psychiatry, 14*(4), 359-375.

van Beijsterveldt, C. E., Groen-Blokhuis, M., Hottenga, J. J., Franić, S., Hudziak, J. J., Lamb, D., . . . Boomsma, D. I. (2013). The Young Netherlands Twin Register (YNTR): longitudinal twin and family studies in over 70,000 children. *Twin Research and Human Genetics, 16*(01), 252-267.

van der Heijden, A. A., Rauh, S. P., Dekker, J. M., Beulens, J. W., Elders, P., M‘t Hart, L., . . . Nijpels, G. (2017). The Hoorn Diabetes Care System (DCS) cohort. A prospective cohort of persons with type 2 diabetes treated in primary care in the Netherlands. *BMJ open, 7*(5), e015599.

Verschuren, W., Blokstra, A., Picavet, H., & Smit, H. (2008). Cohort profile: the Doetinchem cohort study. *International journal of epidemiology, 37*(6), 1236-1241.

Vilhjálmsson, B. J., Yang, J., Finucane, H. K., Gusev, A., Lindström, S., Ripke, S., . . . Won, H.-H. (2015). Modeling linkage disequilibrium increases accuracy of polygenic risk scores. *The American Journal of Human Genetics, 97*(4), 576-592.

Willemsen, G., Vink, J. M., Abdellaoui, A., den Braber, A., van Beek, J. H., Draisma, H. H., . . . Boomsma, D. I. (2013). The Adult Netherlands Twin Register: twenty-five years of survey and biological data collection. *Twin Research and Human Genetics, 16*(01), 271-281.

Wray, N. R., Ripke, S., Mattheisen, M., Trzaskowski, M., Byrne, E. M., Abdellaoui, A., . . . Sullivan, P. F. (2018). Genome-wide association analyses identify 44 risk variants and refine the genetic architecture of major depression. *Nature genetics, 50*(5), 668-681.

Zigmond, A. S., & Snaith, R. P. (1983). The hospital anxiety and depression scale. *Acta Psychiatrica Scandinavica, 67*(6), 361-370.
